# A Randomized Controlled Trial of Local Heat Therapy Versus Intravenous Sodium Stibogluconate for the Treatment of Cutaneous *Leishmania major* Infection

**DOI:** 10.1371/journal.pntd.0000628

**Published:** 2010-03-09

**Authors:** Naomi E. Aronson, Glenn W. Wortmann, William R. Byrne, Robin S. Howard, Wendy B. Bernstein, Mary A. Marovich, Mark E. Polhemus, In-Kyu Yoon, Kelly A. Hummer, Robert A. Gasser, Charles N. Oster, Paul M. Benson

**Affiliations:** 1 Infectious Diseases Division, Uniformed Services University of the Health Sciences, Bethesda, Maryland, United States of America; 2 Infectious Diseases Service, Walter Reed Army Medical Center, Washington (D. C.), United States of America; 3 Department of Clinical Investigation, Walter Reed Army Medical Center, Washington (D. C.), United States of America; 4 Department of Retrovirology, Walter Reed Army Institute of Research, Rockville, Maryland, United States of America; 5 Department of Clinical Trials, Walter Reed Army Institute of Research, Silver Springs, Maryland, United States of America; 6 Clinical Research Management, Hinckley, Ohio, United States of America; New York University School of Medicine, United States of America

## Abstract

**Background:**

Cutaneous *Leishmania major* has affected many travelers including military personnel in Iraq and Afghanistan. Optimal treatment for this localized infection has not been defined, but interestingly the parasite is thermosensitive.

**Methodology/Principal Findings:**

Participants with parasitologically confirmed *L. major* infection were randomized to receive intravenous sodium stibogluconate (SSG) 20mg/kg/day for ten doses or localized ThermoMed (TM) device heat treatment (applied at 50°C for 30 seconds) in one session. Those with facial lesions, infection with other species of *Leishmania*, or more than 20 lesions were excluded. Primary outcome was complete re-epithelialization or visual healing at two months without relapse over 12 months. Fifty-four/56 enrolled participants received intervention, 27 SSG and 27 TM. In an intent to treat analysis the per subject efficacy at two months with 12 months follow-up was 54% SSG and 48% TM (p = 0.78), and the per lesion efficacy was 59% SSG and 73% TM (p = 0.053). Reversible abdominal pain/pancreatitis, arthralgias, myalgias, headache, fatigue, mild cytopenias, and elevated transaminases were more commonly present in the SSG treated participants, whereas blistering, oozing, and erythema were more common in the TM arm.

**Conclusions/Significance:**

Skin lesions due to *L. major* treated with heat delivered by the ThermoMed device healed at a similar rate and with less associated systemic toxicity than lesions treated with intravenous SSG.

**Clinical Trial Registration:**

ClinicalTrials.gov NCT 00884377

## Author Summary

Cutaneous leishmaniasis, a parasitic skin infection transmitted by the bite of a sand fly, can result in chronic skin sores and is estimated to affect more than 1.5 million persons worldwide. While the infection generally heals on its own in months to years, treatment can be expensive and difficult. We compared a heat treatment using the ThermoMed device to an (abbreviated) ten day course of intravenous Pentostam (a pentavalent antimony drug) in a population of U.S. soldiers who acquired their infections in Iraq. We found no statistically significant difference between the two treatments in the healing rate at two months. The heat treatment had less associated toxicity. Heat therapy is a ruggedized, battery operated method that could be adapted to humanitarian situations and less developed health care settings, likely with less cost and side effects than local treatment alternatives.

## Introduction

Cutaneous leishmaniasis is a parasitic infection transmitted by the bite of an infected sand fly, with an estimated worldwide incidence of 1.5 million cases [1]. Generally self resolving within months, infection with *Leishmania major* can leave disfiguring scars or chronic ulcers, usually in areas not covered with clothing. Since 2003, the U.S. military has reported >1,300 cases of cutaneous leishmaniasis in Army soldiers [2]. While systemic use of the pentavalent antimonial sodium stibogluconate (Pentostam, Glaxo Smith Kline, United Kingdom) showed efficacy with treatment responses >90% in cutaneous leishmaniasis, field ready treatment of short duration using limited supplies or equipment was considered preferable [3].

Dermatotrophic *Leishmania* species such as *L. major*, *L. tropica*, and *L. mexicana* are thermosensitive with higher temperatures limiting amastigote replication [4]–[6]. In mice infected with *L. mexicana*, environmental therapy at 37°C for 4–6 weeks was compared to ten day cycles of intralesional meglumine antimoniate. Thermotherapy was associated with more rapid resolution compared to antimonial treatment but both had similar cure rates at 10 weeks (93% and 88% respectively). All untreated control animals had large lesions by week 19 [7].

Heat treatment of leishmaniasis has been delivered by various methods, including hot water soaks, circulating hot water in heating pads, alternating ultraviolet and infrared heat, and ultrasonically induced hyperthermia of the skin [8]–[11]. Clinical trials used several devices which provided localized current from radiofrequency instruments to generate heat in affected tissue [12]–[19]. One of these instruments, ThermoMed Model 1.8, (Thermosurgery Technologies, Inc., Phoenix, AZ) has received 510(K) clearance from the Food and Drug Administration (FDA) for treatment of cutaneous leishmaniasis. Published experience suggests thermotherapy efficacy was similar to or better than intralesional or parenteral pentavalent antimonial treatment, but the studies used differing entry criteria, parasite species, follow-up, and outcome definitions [12], [15]–[19]. Our controlled trial provides detailed information about the course and toxicity in well characterized cases of dermatotrophic *L. major* in “traveling” soldiers without prior immunity. Our subjects left the endemic region by the time of treatment and were adherent with follow-up, thereby eliminating factors that plagued prior studies.

## Methods

### Participants/Study Location

The trial was performed at Walter Reed Army Medical Center (WRAMC) in Washington, DC in 2004–6. Eligible participants were Department of Defense health care beneficiaries with parasitologically confirmed cutaneous leishmaniasis. Based on travel history, all participants were likely infected in Iraq or Kuwait. All were treatment naive. Randomization criteria included *L. major* species confirmation. Exclusionary criteria were >20 lesions, pregnancy, lactation, hypersensitivity to pentavalent antimonials or local anesthetics, serious medical illness, lesion in proximity to mucous membranes, face, or cartilage, implanted metallic devices, or unwillingness to avoid procreation for at least two months. The protocol received Institutional Review Board approval at Walter Reed Army Medical Center and all participants provided written informed consent. A sample size of 27 participants per treatment group was planned, assuming a 73% cure rate of TM [12], 99% for SSG [20], controlling for a probability of a Type I error at alpha  = 0.05 and was predicted to have 80% power to determine a 26% difference in outcome.

### Study Interventions

This controlled trial compared 10 days of 20 mg/kg/day intravenous sodium stibogluconate (SSG, GlaxoSmithKline, United Kingdom) to one treatment session using the ThermoMed Model 1.8 device (TM). SSG was infused over 10–50 minutes for a total of 10 doses. Participants randomized to thermotherapy received oral antibiotics for secondary bacterial infections of the leishmaniasis lesion(s) prior to treatment (SSG arm participants were treated concurrently). Prior to thermotherapy, each lesion was cleansed, anesthetized, moistened, and overlying eschar was removed. Two trained physicians performed TM treatments. The TM probe was placed on the skin, covering the lesion with 50°C TM treatments applied for 30 seconds in a grid fashion extending 4mm into border skin. The lesion size determined the number of applications. Each lesion was then covered with a dressing (Coverlet, Beiersdorf-Jobst, Wilton CT) that was changed daily. Participants were seen daily for 10 days with physical examination, laboratory testing (complete blood count, creatine phosphokinase, amylase, lipase, complete metabolic profile), and electrocardiograms at baseline, days 3, 7, 10. Interview follow-up by telephone, email, letter, or infrequently in person, occurred at 2, 6, and 12–24 months post treatment. Photographs were taken at baseline, day 10, and requested with 2, 12 month follow-ups. Sequential photographs were independently assessed by blinded leishmaniasis experts, who were clinicians experienced in the treatment of CL, with a tiebreaker assessment when needed. Participants with clinical failure at 2 months were offered crossover treatment.

### Objectives

The primary study objective was to compare efficacy of local thermotherapy with the TM device to a 10 day course of parenteral SSG, (IND# 14150), to treat cutaneous *L. major* (2 months post treatment). Secondary objectives included comparison of the treatment toxicity profiles.

### Study Outcome Measures

Clinical cure was defined as complete epithelialization or visually healed at 2±1 month after completion of therapy and no reactivation in 12 months after the start of treatment. Clinical failure was less than complete epithelialization or visually not healed at 2±1 month after treatment completion. Relapse failure was defined as skin lesion persistence at the treatment site or elsewhere in the period up to 12 months after start of therapy, regardless of appearance at 2±1 month after treatment completion. The overall treatment responses are reported as determined by: subject, blinded expert photograph review, and consensus response where photograph review was the prioritized outcome but subject assessment was used when photographs were not provided or were inadequate for review.

### Randomization

The statistician (RH) generated the randomization plan in blocks of 4 subjects using www.randomization.com. The research pharmacist made assignments using the randomization plan in sequential order. The allocation sequence was unavailable to investigators until completion of the trial.

### Parasitologic Confirmation

Diagnosis was confirmed prior to enrollment using skin scrapings or biopsy from one characteristic lesion per subject. Histology was reviewed at the Armed Forces Institute of Pathology, Washington, DC. *Leishmania* culture and genus/species PCR were done at the Walter Reed Army Institute of Research, Silver Spring, MD. Speciation was determined by isoenzyme analysis of positive cultures and a glucose phosphate isomerase (GPI) based RT-PCR assay for *Leishmania* genus PCR positive samples [21].

### Statistical Methods

Subject data were entered into Access (Microsoft) software and analyzed in SPSS (Windows v17, SPSS Inc., Chicago, IL). Groups were compared using Fisher's exact test and the Wilcoxon rank sum test. Logistic regression and the Mantel Haenszel test were used to examine the effect of multiple risk factors on healing. Serial laboratory assessments were compared using repeated measures analysis of variance. Data not satisfying assumptions of normality were logarithmically transformed. All p values are two-sided and p<0.05 was considered statistically significant. Study data were analyzed in an intent to treat (ITT) basis with missing data handled as missing. The four treatment crossover subjects were considered failed in the analysis, the twelve month outcome was not evaluable given multiple treatment modalities.

## Results

**Figure 1:**
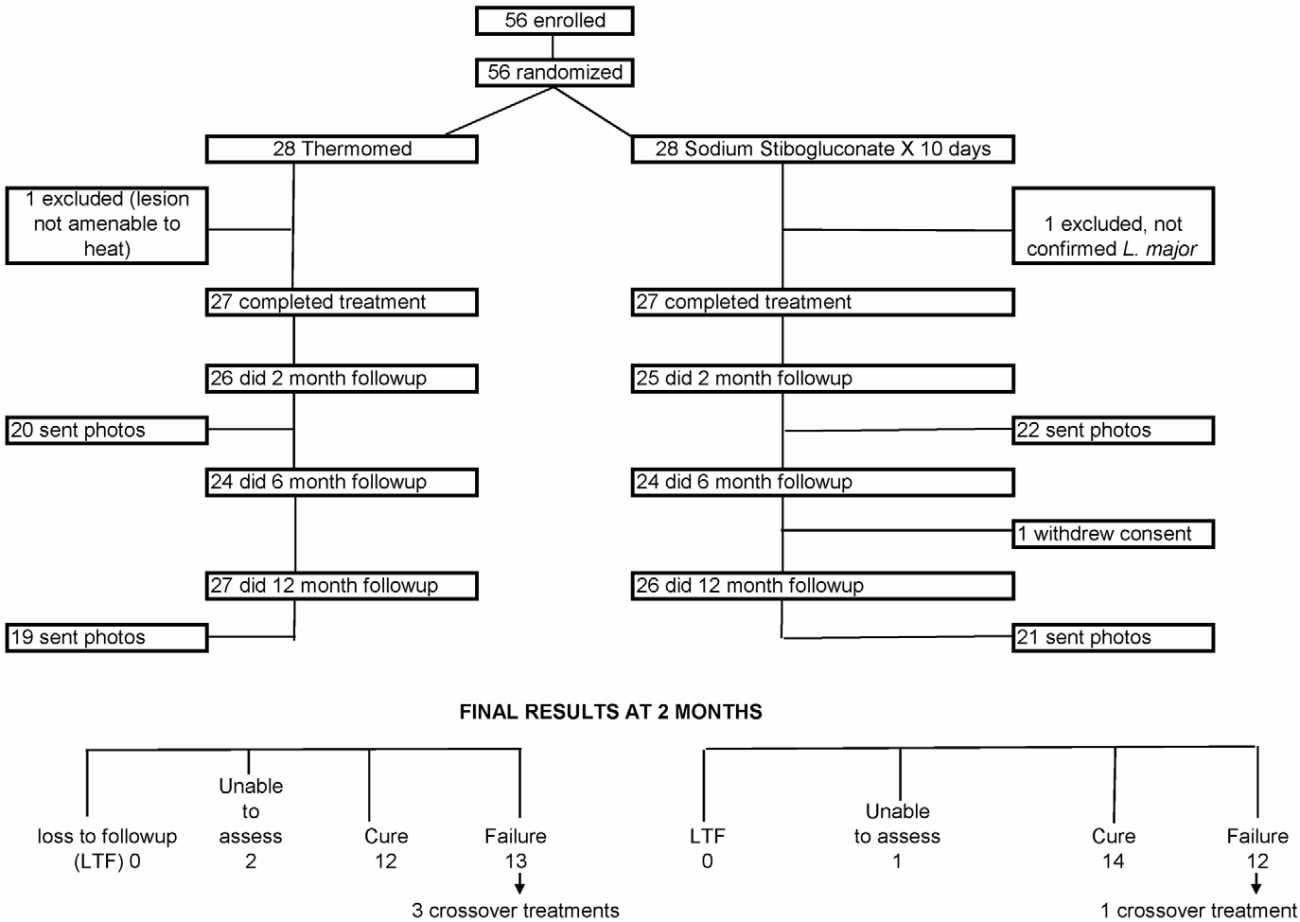
Study flow diagram.

We enrolled 56 participants but two were subsequently excluded: one lacked confirmed speciation and one for olecranon bursitis under a lesion which might have been associated with excessive burn injury. Thus, 27 participants in each arm completed study prescribed treatment. Figure 1 shows good adherence with study follow-up visits. End points and follow-up data were available for all but one SSG treated participant who withdrew from follow-up after the 6 month visit. Participants receiving crossover treatment and the withdrawn subject were unable to be assessed for the 12 month outcome (3 in TM, 2 in SSG), although all eligible were contacted (n = 4) and endorsed being healed.

The baseline demographic characteristics (Table 1) were similar between treatment arms with the exception of lesion location. In the SSG treatment group there was a higher number of arm and chest lesions and fewer neck, leg, and back lesions. All treated participants had parasitologic confirmation of *L. major* infection.

**Table 1:** Baseline Demographic Characteristics Compared by Treatment.

	TM	SSG	p value
**Characteristic per patient**	n = 28	n = 28	
Median age in years (range)	25 (20–53)	24 (18–57)	0.16
Male gender (%)	28 (100)	27 (96)	0.99
Race			
Caucasian (%)	15 (54)	18 (64)	0.84
African American (%)	7 (25)	5 (18)	
Hispanic (%)	4 (14)	4 (14)	
Other (%)	2 (7)	1 (4)	
Median number of lesions (range)	2 (1–14)	3 (1–17)	0.74
Median duration lesion in days, (range)	126 (45–231)	138 (50–270)	0.75
Amastigotes present (%)	22 (79)	18 (64)	0.38
Culture isoenzyme *L. major* * (%)	12/23 (52)	12/24 (50)	0.99
*L. major* species PCR positive	28 (100)	27 (96)	0.99
Complicated leishmaniasis** (%)	5 (18)	8 (29)	0.53
**Characteristic per lesion**	n = 94	n = 97	
Median size of lesion mm^2^ (range)***	155 (9–1014)	110 (9–1720)	0.14
Location of lesion (%)			0.002
Head & Neck	11 (12)	6 (6)	
Arms	40 (43)	65 (67)	
Legs	27 (29)	14 (14)	
Back	11 (12)	3 (3)	
Chest	5 (5)	9 (9)	

*Not all subjects had parasite culture done so denominator given in table.

**Presence of significant regional adenopathy or subcutaneous nodules.

***Size is length x width of skin lesion.

**Figure 2:**
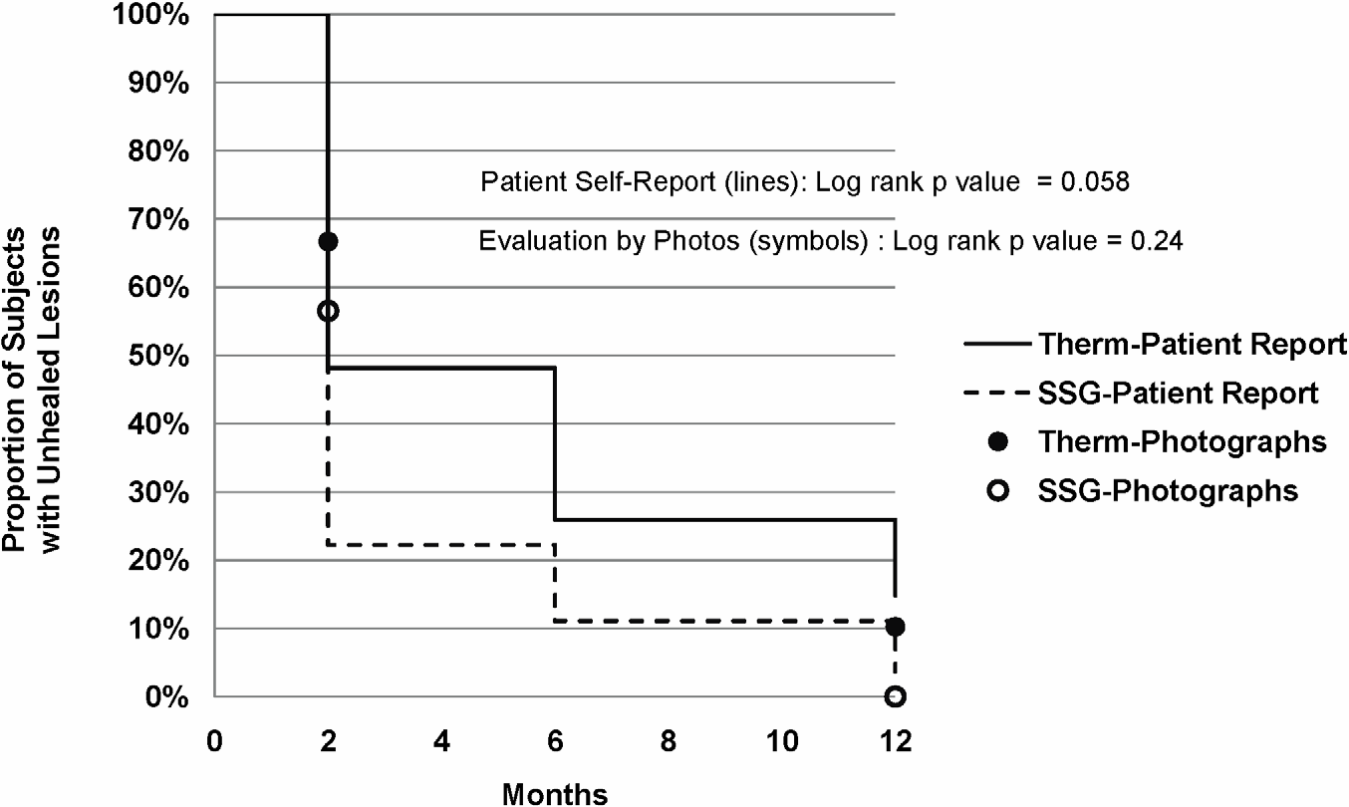
Time to healing by patient self report or by photographs.

In an ITT analysis at two months after the completion of treatment, 12/25 evaluable TM participants (48%) and 14/26 SSG treated participants (54%) were assessed as clinically cured with complete and durable healing of all lesions (p = 0.78, odds ratio: 0.79, (95% CI: 0.26–2.38)). At 2 months, 63 TM treated lesions (73%) versus 50 SSG treated lesions (59%) were assessed as clinically cured (p = 0.053, odds ratio: 1.92 (95% CI: 1.01–3.65)). There were two relapse failures. Relapses occurred prior to the six month follow-up and in the TM arm included activation/ulceration at a site not treated, subcutaneous nodules forming around the treated area, or breakdown within the edge of the TM treated area. After the two month follow-up, three persons in the TM assigned arm crossed over to SSG and one person in the SSG assigned arm was subsequently retreated with TM. Other participants with clinical failure at 2 months elected for no further treatment. The survival analysis of time to healing for the two treatment arms (Figure 2) suggested that participants in the SSG treated arm appreciated a more rapid time to healing as compared to thermotherapy treated (p = 0.058) when healing was based solely on patient evaluation. This difference was not found in the analysis using blinded reading of photographs where the efficacy by treatment was statistically similar (p = 0.24).

**Figure 3:**
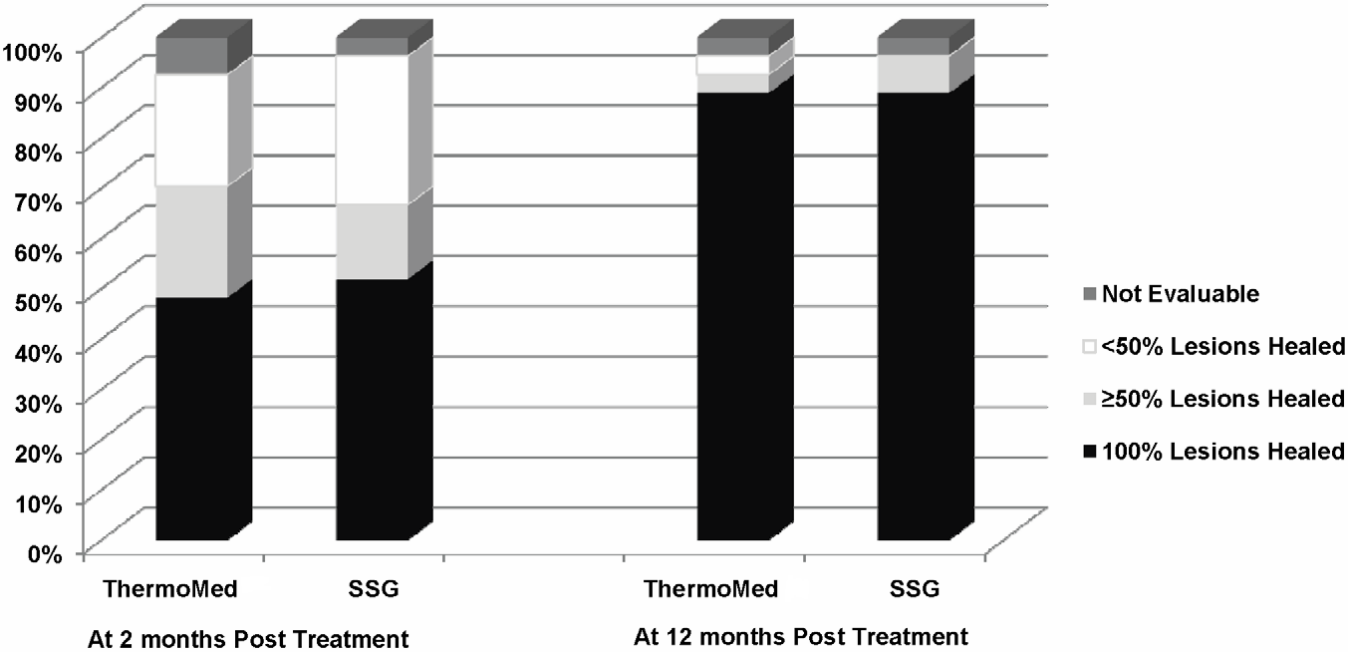
Consensus treatment efficacy at two and twelve months follow-up.

The demographics of participants with clinical cure at 2 months versus failure (Table 2) suggested no difference in race, duration of lesion, lesion size, or number of TM treatments/lesion. Subjects with clinical cure were 3 years younger (p = 0.019). There was a difference in the participant response between the two TM operators (p = 0.019), but not a per lesion response difference, (p = 0.38). The operator with the higher failure rate treated subjects with more lesions (median = 3, range: 1 to 14) compared to a median of 2 (range: 1 to 4) for the other operator (p = 0.06). Among those participants with clinical failure, the median number of lesions was higher (p = 0.026) and lesion location also differed with more arm, chest involvement in those failing (p = 0.001). When the number of lesions and age were included in a multivariate analysis, the difference between treatment groups was not significant (p = 0.85, odds ratio: 0.89 (95% CI: 0.27–2.91)). When location was taken into account in the analysis of lesions, treatment groups did not differ significantly (p = 0.30, odds ratio: 1.44 (95% CI: 0.72–2.86)). Complete cure at two months after completion of treatment was a very stringent outcome assessment, so we compared clinical outcome where participants were graded as <50% lesions healed, ≥50% lesions healed and 100% lesions healed. This demonstrated no significant differences in efficacy between treatment arms at the 2 or 12 month follow-ups (Figure 3).

**Table 2:** Characteristics of those healed versus failed at two months.

	Clinical Cure	Clinical Failure	p Value
**Characteristic per patient**	n = 26	n = 25	
Median age in years (range)	23 (19–38)	26 (18–57)	0.019
Race			0.99
Caucasian (%)	15 (58)	16 (64)	
African American (%)	6 (23)	5 (20)	
Hispanic (%)	3 (11)	3 (12)	
Other (%)	2 (8)	1 (4)	
Median # lesions (range)	2 (1–14)	3 (1–17)	0.026
Median duration lesion in days (range)	115 (45–270)	138 (55–196)	0.82
ThermoMed operator/patient			0.015
Dr. X (n = 15)	4 (33)	11 (85)	
Dr. Z (n = 10)	8 (67)	2 (15)	
**Characteristic per lesion**	n = 113	n = 63	
Median size of lesion in mm^2^ (range)	130 (9–1720)	135 (12–750)	0.62
Location of lesion			0.001
Head & Neck (%)	9 (8)	6 (10)	
Arms (%)	53 (47)	40 (69)	
Legs (%)	34 (30)	3 (5)	
Back (%)	10 (9)	3 (5)	
Chest (%)	7 (6)	6 (10)	
**ThermoMed treated lesions only**			
ThermoMed operator/lesion			0.38
Dr. X (n = 69)	48 (76)	20 (87)	
Dr. Z (n = 19)	15 (24)	3 (13)	
Median no. of ThermoMed Tx/lesion	2 (0–13)	3 (1–7)	0.076

A secondary objective was comparison of toxicity profiles. Daily physician evaluations occurred during treatment with frequent laboratory and electrocardiogram evaluation. There were seven serious adverse events, four in the TM arm and three in the SSG arm. All were assessed as no or remote relationship to study treatment, consisting of a basal cell carcinoma diagnosis prior to treatment and hospitalizations during the follow-up period for gastroenteritis, elective repair of the left acromioclavicular joint, pneumonia, surgery for rectal carcinoid, inflammatory bowel disease, and trauma from a motor vehicle accident. Serial laboratory values over the 10 day treatment, segregated by treatment modality, show statistically significant decreases in white blood cells, hematocrit, platelets, and increases in amylase, lipase, ALT, AST in those receiving SSG (Table 3). Of note, changes in creatinine, corrected QT interval, and creatine kinase (CK) were similar.

**Table 3:** Median laboratory values over time, compared by treatment group.

Median Value (range)	Baseline	Day 3	Day 7	Day 10	p value*
WBC in TH/CUMM					0.017
TM	7.2 (4.0,12.5)	6.8 (3.6, 11.2)	7.0 (3.8, 12.2)	6.4 (3.9, 10.3)	
SSG	7.9 (3.5, 12.4)	5.9 (3.0, 9.0)	5.5 (2.9, 12.0)	4.9 (3.1, 10.6)	
Hematocrit %					<0.001
TM	44.6 (36.8, 56.6)	45.4 (36.9, 53.5)	44.8 (38.3, 52.6)	45.9 (40.9, 50.9)	
SSG	44.3 (35.4, 55.3)	44.1 (37.7, 51.5)	44.0 (38.1, 51.0)	43.1 (37.6, 47.7)	
Platelets TH/CUMM					<0.0005
TM	250 (171, 353)	233 (170, 323)	237 (167, 302)	236 (181, 291)	
SSG	250 (179, 333)	229 (180, 310)	206 (134, 289)	196 (133, 294)	
Amylase U/L					<0.0005
TM	51 (<30, 101)	48 (30, 104)	52 (30, 93)	52 (33, 88)	
SSG	50 (<30, 107)	127 (34, 420)	152 (<30, 511)	143 (<30, 348)	
Lipase U/L					<0.0005
TM	72 (29, 254)	61 (29, 224)	75 (28, 212)	70 (19, 253)	
SSG	76 (26, 204)	512 (95, 2479)	587 (103, 6009)	515 (97, 2385)	
Creatinine mg/dL					0.92
TM	1.0 (0.8, 1.2)	1.0 (0.9, 1.2)	1.0 (0.8, 1.2)	1.0 (0.8, 1.2)	
SSG	1.0 (0.7, 1.3)	1.0 (0.8, 1.3)	0.9 (0.7, 1.4)	0.9 (0.7, 1.3)	
CK U/L					0.91
TM	103 (60, 17763)	102 (57,1475)	117 (54, 522)	110 (57, 572)	
SSG	78 (52, 220)	97 (39, 453)	90 (34, 206)	78 (34, 404)	
ALT U/L					<0.0005
TM	30 (22, 86)	29 (19, 86)	32 (17, 73)	30 (21, 77)	
SSG	27 (16, 61)	27 (14, 67)	46 (22, 400)	71 (28, 305)	
AST U/L					<0.0005
TM	22 (18, 187)	25 (17, 70)	25 (17, 35)	27 (16, 55)	
SSG	23 (13, 42)	23 (14, 52)	37 (18, 285)	52 (26, 135)	
QTc ms					0.24
TM	403 (369, 437)	403 (319, 440)	401 (378, 457)	402 (372, 457)	
SSG	399 (377,450)	402 (377, 438)	403 (368, 447)	403 (376, 438)	

*p Values represent interaction of treatment and time (e.g. a significant p value indicates that the effect of the treatment over time was significantly different).

After treatment initiation, there were five lesion-related wound infections (19%) in the TM treated arm and one (4%) in the SSG treated group (p = 0.19). In the TM arm, the median time to wound infection after treatment was 10 days (range 3–75). Lesion reactions associated with TM included blister 25 (93%), oozing 21 (78%), and keloid 2 (7%). Participant description of their lesions showed no qualitative difference between treatment arms at the 12–24 months follow-up. Chest pain, abdominal discomfort, diarrhea, nausea, vomiting, arthralgias/myalgias, rash, headache, dizziness, fatigue, and pyrexia were more common in the SSG treated arm, (Table 4). Among SSG treated participants, 82% developed elevated amylase or lipase at some time versus none in the TM treated arm. 46% SSG and 7% TM treated developed elevated ALT/AST. CK was increased over baseline values in 25% SSG and 32% TM.

**Table 4:** Common adverse events compared by treatment arm*.

Adverse Event	TM	SSG	p value
	(n = 27)	(n = 27)	
Cardiac Disorders n (%)	10 (37)	15 (56)	0.28
EKG changes	10 (37)	14 (52)	0.41
Chest pain	0 (0)	3 (11)	0.24
Palpitations	0 (0)	1 (4)	0.99
Gastrointestinal n (%)	3 (11)	18 (67)	<0.0005
Abdominal discomfort/pain	1 (4)	12 (44)	0.001
Diarrhea	2 (7)	7 (26)	0.14
Nausea	2 (7)	8 (30)	0.076
Vomiting	0	3 (11)	0.24
Infection n (%)	9 (33)	6 (22)	0.54
Wound infection	5 (19)	1 (4)	0.19
*Leishmania* lesion reaction n (%)			
Blister	25 (93)	0 (0)	<0.0005
Erythema	7 (26)	1(4)	0.05
Keloid	2 (7)	0 (0)	0.49
Oozing	21 (78)	2 (7)	<0.0005
Musculoskeletal n (%)	5 (19)	18 (67)	0.0010
Arthralgias	3 (11)	16 (59)	<0.0005
Myalgias	2 (7)	12 (44)	0.004
Nervous system n (%)	5 (19)	17 (63)	0.002
Headache	3 (11)	12 (44)	0.014
Dizziness	2 (7)	4 (15)	0.67
Respiratory n (%)	3 (11)	6 (22)	0.47
Cough	0 (0)	2 (7)	0.49
Dyspnea	1 (4)	1 (4)	0.99
General n (%)	5 (19)	23 (85)	<0.0005
Fatigue	5 (19)	18 (67)	0.001
Pyrexia	0 (0)	4 (15)	0.11
Skin n (%)			
Rash	1 (4)	4 (15)	0.35
Serious adverse events n (%)	4 (15)	3 (11)	0.99

(All are no or remote relationship).

*Each event counted once per participant.

## Discussion

Standard treatment practice in the U.S. for cutaneous *L. major* includes no treatment (generally for <5 uncomplicated skin lesions), use of investigational drugs including SSG, miltefosine, paromomycin, or off label use of azoles or amphotericin products. These present an array of adverse effects to consider in each individual with a cosmetically concerning/scarring dermal infection generally not associated with systemic complications.

Our results confirm that a single heat treatment of lesions with TM was as effective as 10 days of intravenous SSG at 20mg/kg/day in *L. major* infection in American soldiers. Our primary endpoint of healing at two months with no reactivation over 12 months showed 48% TM and 54% of SSG treated participants achieved a clinical cure. Additionally, per lesion analysis showed 73% of TM treated lesions and 59% of SSG treated lesions at 2 months follow-up were clinical cures (p = 0.053). We had excellent adherence; all receiving study treatment completed the course with daily physician follow-up for ten days. Unexpectedly, interview responses from participants regarding outcome at 2, 6, 12–24 months suggested a perception that SSG was associated with more rapid healing (p = 0.058). This was not corroborated by the expert (blinded) committee assessment of lesion photographs. We speculate a perception bias developed among participants that TM was a less potent treatment (SSG is a standard treatment at WRAMC and does not initially enlarge the lesion like TM). Participants generally endorsed no perceived cosmetic outcome difference between the treatments at the latest follow-up.

We monitored toxicity of TM and SSG with laboratory and electrocardiogram testing, daily physician assessments for 10 days after treatment and long follow-up (12–24 months). The thermotherapy arm provided insight into the differential frequency of adverse effects of a 10 day course of intravenous pentavalent antimony. Systemic effects including pancreatitis (67%), arthralgias, myalgias (59%), headache (44%) and fatigue (67%) were more likely in the SSG treated group and local effects of lesion blistering (93%), oozing (78%), and erythema (26%) were more likely in the TM (which causes a burn) treated arm. Interestingly, there was no statistical difference in electrocardiogram findings, including changes in QTc, between the two treatment groups. Compared to the typical SSG regimen of 20 days in a similar military population, the magnitude of change in laboratory values was lower in the 10 day SSG infusion course [3].

Previous studies of thermotherapy in Old World cutaneous leishmaniasis have suggested reasonable but variable efficacy. A small observational study in U.S. soldiers showed 88% efficacy of TM treatment with a 6 months follow-up [16]. A large randomized trial in Afghanistan of presumed *L. tropica* using the ThermoMed device found 69% cure at 100 days follow-up and lesser efficacy (45%) with intramuscular SSG (Albert Davis Ltd, India) with a maximum dose of 850 mg per day [17]. In Iran (presumed *L. major* species) a different radiofrequency heating device was used in four weekly sessions compared to intralesional meglumine antimoniate with 6 month efficacy of 81% for thermotherapy and 55% for intralesional antimonial [18]. Studies of thermotherapy in New World cutaneous leishmaniasis have also suggested efficacy: 73% in Guatemala [12], 90% at eight weeks in Mexico [14], 71% in Brazil at 28 days [15], and 100% per protocol but 19% in ITT analysis in Colombia at 100 days [19].

Our study has an early outcome measure at 2 months after the start of treatment, although information at 6 months was also obtained. It is difficult to identify another OWCL study where similar species (often mixed/not specified *L tropica* and *L. major*) and outcome definition were applied at 2 months. We tested a shorter course using full dose intravenous SSG (no upper limit to the daily weight based dose) which has not been studied head to head in a randomized trial of other agents. While the standard duration of intravenous SSG is 20 days, a previous study at our center suggested similar efficacy using a 10 day course at 6 month follow-up, with less toxicity [21]. From 2003–6, in 141 *L. major* patients treated at our center we found 92% clinical cure with SSG 10 versus 95% in SSG 20 patients (n = 273) at six months follow-up [personal communication Aronson]. Importantly, this thermotherapy study findings suggest that a single heat treatment of cutaneous leishmaniasis with a FDA ‘approved’ device suitable for resource limited areas has similar healing rates to SSG for 10 days. We further described the efficacy and lower toxicity seen with short duration intravenous SSG therapy in *L. major* infection.

The strengths of our study include a randomization, similar detailed assessments of both treatment arms, prolonged follow-up, excellent adherence, parasitologic species confirmation, and rigorous outcome assessment with treatment blinded experts reviewing lesion photographs supplemented with participant's impression. A universal measure of healing in Old World cutaneous leishmaniasis is lacking but the standard timepoint seems to be 3 months after treatment [22]. We chose an earlier timepoint to maximize our observation of the effect of our interventions in a self healing infection. Regarding study limitations, our study lacks a placebo arm to address the self healing observed with time and variably described as 10–68% in a systematic review in *L. major* [23]. A Cochrane review suggested *L. major* self heals in 2–3 months, but may persist up to 5 years [22]. In our study, the median duration of skin lesions prior to treatment exceeded 120 days; there likely was a referral bias for more persistent and severe *L. major* infection being sent back to the U.S. for treatment. Regarding diagnosis, only one lesion was sampled per participant and unresponsive skin lesions may not always have been cutaneous leishmaniasis, however in a randomized clinical trial this is unlikely to have a differential effect. The generalizability of our results is limited to *L. major* lesions excluding the face, of moderate size (mean 205 mm^2^), in otherwise healthy men who constituted our study population. The outcome assessment was restricted by the lower quality of some and incomplete provision of returned photographs as well as subjective information relayed by the participants rather than in person evaluations during the follow up.

The clinical implications of our study are that a single thermotherapy treatment showed similar healing rates and was as well tolerated as a 10 day infusion of SSG for *L. major* infection. Thermotherapy devices are portable, multi-use, and easy to operate in resource limited environment. This contrasts to the time, labor, and cost involved using an intravenous investigational new drug such as SSG under FDA regulations which required transfer to the U.S. for our subjects. Recognizing that *L. major* often results in a self healing infection, the choice of treatment intervention should weigh patient wishes, and consider the number, location, duration, size of lesions, and signs of local dissemination. Thermotherapy is generally well tolerated and may limit the size of subsequent scar. The effect is local so use in disseminated infection is not recommended. Large numbers of lesions and very large lesions require a significant amount of time to treat with the device. To mitigate the risk of secondary wound infection of the burn, others have used topical bacterial ointment during the initial post treatment period [16]. Analyses have not found antimonial treatment of cutaneous leishmaniasis as cost-effective. While thermotherapy cost has not been included in these models, it is likely to be cost-effective in endemic areas [17],[24],[25]. Future research could assess whether thermotherapy treated areas showed persistent *Leishmania* organisms as seen after clinical healing post SSG; if not, then the implication of lessened risk of later reactivation but also the role of potentially lessened protection from reinfection might be better understood [26],[27].

## Supporting Information

Checklist S1 CONSORT checklist.
(0.19 MB DOC)

Protocol S1 Trial protocol.
(2.10 MB PDF)
