# Supplementary material for: A Randomized Controlled Trial of Local Heat Therapy Versus Intravenous Sodium Stibogluconate for the Treatment of Cutaneous Leishmania major Infection
Source: PLoS Negl Trop Dis. 2010 Mar 9;4(3):e628. doi: 10.1371/journal.pntd.0000628 (PMC2834752; doi:10.1371/journal.pntd.0000628)
Supplement: Protocol S1 — Trial protocol. (2.10 MB PDF) [file pntd.0000628.s002.pdf]

WU#: 03-19008
Revised per HUC 24 June 2003
Revised per CIC 5 June 2003
Revised per hsrrb prereview 24 August 2003
Revised per hssrrb review sent 23 Sep 2003
Revised 19 Dec 2003 for USAMMDA
Revised 6 January 2004 for HUC

MCHL-MI

**DATE:** 6 January 2004

**MEMORANDUM FOR CHIEF, DEPARTMENT OF CLINICAL INVESTIGATION, WALTER REED ARMY MEDICAL CENTER**

**SUBJECT: Application and Request for Approval of Clinical Investigation Study Proposal** *(Revision 6)*

1. **PROTOCOL TITLE**: A Phase I/II randomized comparison of localized heat therapy versus Sodium Stibogluconate (Pentostam ®) for the treatment of Old World cutaneous leishmaniasis

2. **PRINCIPAL INVESTIGATOR:**


Naomi E. Aronson Colonel Medical Corps (COL MC)
Director, Infectious Diseases Division
Department of Medicine Uniformed Services University (USUHS)
Telephone (Phone): (301) 295-3621
Facsimile (Fax) Number: (301) 295-3557
naronson@usuhs.mil


3. **ASSOCIATE INVESTIGATORS:**


Gail Robinson ,Registered Nurse (RN)
Head Nurse, Leishmania Treatment Center
Department of Medicine
Phone (202) 782- 8720
Fax: (202) 782-3765

Glenn Wortmann, Lieutenant Colonel (LTC) MC
Associate Program Director, Infectious Diseases
Department of Medicine
Phone: (202) 782-8684

Charles Oster COL MC
Chief, Infectious Disease Clinic
Department of Medicine
Phone: (202) 782-8695

Paul Benson COL MC
Staff physician, Dermatology Service
Department of Medicine
Phone: (202) 782-6175

Mark Polhemus, Major (MAJ) MC
Fellow, Infectious Diseases
Department of Medicine

Phone (202) 782- 8711

Mary Marovich, Medical Doctor (MD) Diplomate Tropical Medicine and Hygiene (DTMH)
Combined United States(US) Military Human Immunodeficiency Virus (HIV) Research Program
13 Taft Court
Rockville, MD 20850
Phone (301) 251-8337

Wendy Bernstein, Colonel Medical Corps
Walter Reed Army Institute of Research
1600 E. Gude Dr.
Rockville, MD 20850
Phone (301) 251-5087

## 4. COLLABORATING PERSONNEL:

**PROTOCOL SPONSOR REPRESENTATIVE:**
MAJ David Shoemaker
Project Manager
United States Army Medical Materiel Development Activity (USAMMDA)
622 Neiman Street
Ft. Detrick, Maryland (MD) 21702
Phone: (301) 619-7871

**Role**: Will serve as agent for sponsor (Office of the Army Surgeon General[OTSG] /USAMMDA), also arrange and provide for supply of Sodium stibogluconate and protocol monitoring

Sheila Peel PhD
Director De Novo Microarrays/Flow Cytometry
Department of Molecular Diagnostics/Pathogenesis
Walter Reed Army Institute of Research
1600 E. Gude Drive
Rockville MD 20850
Phone (301) 251-8346

## 5. MEDICAL MONITOR:

Kent Kester LTC(Promotable) MC
Chief, Clinical Trials Section
Division of Communicable Diseases and Immunology
Walter Reed Army Institute of Research (WRAIR)
Phone: (301) 319- 9234
Fax: (301) 319-9012

## 6. ABSTRACT:


Primary Objective:

1. Assess efficacy of local heat using Thermosurgery Technologies, Inc. (TTI) ThermoMed™ device versus sodium stibogluconate (SSG) IV 20mg/kg/d for 10 days, for treatment of cutaneous *L. major* infection (cure at 2 months after treatment started). Both treatment arms will be considered experimental treatments.

Secondary Objectives:

1. Determine efficacy of heat versus SSG in clinical response of all *Leishmania major (L. major)* skin lesions at 12 months after treatment initiated
2. Compare toxicity profile of heat versus SSG treatment
3. Determine feasibility of *L. major* species - specific polymerase chain reaction as a rapid diagnostic device in the context of a treatment trial
4. Determine the immune response to *Leishmania* in both treatment arms:

Study Design:

A 2 arm, randomized trial of local heat therapy using ThermoMed™ device versus 10 days of systemic 20mg/kg/d sodium stibogluconate in the treatment of cutaneous old world Leishmaniasis. 27 patients per treatment arm, study duration 12 months.

Methods:

Arm A: Patients will receive one treatment per lesion with ThermoMed™ device 50°C for 30 seconds

Arm B: Patients will receive daily infusions of sodium stibogluconate 20mg/kg/day for 10 doses


## 7. OBJECTIVES:

**Primary objective:**

Assess if local heat therapy using the TTI ThermoMed ™ device is equivalent in efficacy to 10 days of parenteral Sodium stibogluconate (SSG), IND( Investigational New Drug) #14150, for treatment of cutaneous infection with *Leishmania major* (primary endpoint is cure of infection at two months after treatment completed)

**Secondary objectives:**

- Determine the efficacy of heat versus SSG in clinical response of all *Leishmania* skin lesions at 12 months after treatment initiated (secondary endpoint is combined clinical cure and intermediate response in each arm)
- Compare the toxicity profiles of heat versus parenteral SSG therapy
-Establish the feasibility of a *L. major* species specific polymerase chain reaction as a rapid diagnostic device in the context of a treatment trial (compare to culture/ isoenzyme analysis)
- Evaluate the immune response to *Leishmania* before, at 10 days, and six months in recipients of localized heat therapy versus systemic SSG

## 8. MEDICAL APPLICATION:

Leishmaniasis is a protozoal disease transmitted by sandflies. *Leishmania major* is common in many parts of the world including Southwest Asia and the Middle East. Infected humans may develop cutaneous disease with *L. major* which can be somewhat resistant to antimonial treatment. In most, *L. major* is a self limited infection that resolves without specific treatment in 6-18 months. Therefore, an alternative to antimony that works as well but has less toxicity, does not require IND parenteral therapy, and would not require transfer to tertiary care, would be very useful to the US military.

Leishmaniasis is a medical threat for military soldiers assigned in endemic areas. *L. major* has been identified in individuals involved in the Persian Gulf War and those deployed to Kuwait and Afghanistan since then. It is a potential medical threat for future conflicts with Afghanistan, Iraq, Kuwait, Iran, Syria, and Saudi Arabia having some of the highest prevalence of *Leishmania major* infection in the world.

## 9. BACKGROUND AND SIGNIFICANCE:

Pentavalent antimonials (Pentostam, Wellcome Foundation, United Kingdom (UK) and Glucantime, Rhone Poulenc, France) have been used to treat leishmaniasis for more than 50 years. Neither of these drugs is licensed for commercial use in the United States, likely because of limited use. Worldwide, there is a great deal of experience and use of these agents. Sodium stibogluconate is a pentavalent antimony complexed to carbohydrate whose exact structure and mechanism of action is not known.(2) It is provided as a 100 mg antimony/ml solution that contains a preservative, m-chlorocresol. Most of the dose is excreted by the kidneys within 24 hours.

In 1984 World Health Organization recommended the daily dose of antimony (Sb) in treatment of visceral leishmaniasis be increased to 20 mg/kg/day. A randomized controlled trial(3) of 40 patients with American cutaneous leishmaniasis (ACL) found 100% cure rates with 20 mg/kg/day Sb for 20 days but only a 76% cure if 10 mg/kg/day for 10 days was used. A comparison (4) of three treatment schedules in 36 patients with cutaneous leishmaniasis (single rapid infusion, continuous 24 hour infusion, or every eight hour doses) found no advantage over using once daily dosing. A review (5) of the controlled trials of Sodium stibogluconate concludes that a recommended course of therapy is 20 mg/kg/day with no upper limit to dose for 20 days for cutaneous leishmaniasis and 20 mg/kg/day for 28 days for visceral or mucocutaneous leishmaniasis. The Pentostam® package insert suggests 10-20 mg/kg/day with a maximum dose of 850 mg for a minimum of 20 days. The CDC investigators brochure supports the use of 20mg/kg/day with no upper limit to dosage. We recently published our cutaneous leishmaniasis treatment experience comparing Sodium stibogluconate 20 mg /kg for 10 versus 20 days and found 100% of the 10 day group were cured. In this study 15% were *Leishmania major* infections.(6)

Detailed toxicity data for the 20mg/kg/day dose is provided by several studies (1,7-14). Percentages from our WRAMC experience are included here. Subjective musculoskeletal complaints are common (58%), as well as elevated hepatocellular (67%) and pancreatic enzyme levels(97%) and nonspecific electrocardiogram changes (T wave changes). These side effects are usually reversible. Conventional alternative therapies to pentavalent antimony (amphotericin B and pentamidine) are also associated with toxicity and reserved for patients where antimonial therapy is not effective. Other Sodium stibogluconate toxicity includes headache (22%), rash (9%), thrombocytopenia, depression of various hematologic cell lines (44%), phlebitis,

anaphylaxis, inflammation around lesions (in the case of nasopharynx and tracheal involvement with mucocutaneous leishmaniasis this can be severe), transient coughing after infusion. Other associated symptoms include anorexia, malaise, myalgia, abdominal pain, headache, lethargy, sweating, vertigo, facial flushing, initial worsening of skin lesions, epistaxis, jaundice and peripheral neuropathy. In our above mentioned 10 versus 20 days study, the adverse events were significantly decreased in the cohort receiving the 10 days versus 20 with myalgias in 42% ( versus 68%), less chemical pancreatitis, and hematological parameter decreases. (6)

Laboratory investigation showed that *Leishmania* infection of mouse peritoneal macrophages was optimal at 35° C and that at 39° C several strains were completely destroyed.(15) In a human macrophage system, *L. tropica* grew better at 35°C than 37°C, and was destroyed at 39°C. (16) Elevated environmental temperatures were reported to be curative in *Leishmania enrietta* infections in guinea pigs. (17) Neva (18) reported success using local heat treatment (heating pad with warm water (39-41° C) circulating through it for > 35 hours ) in 3/5 patients with diffuse cutaneous leishmaniasis (L. mexicana) , however in 3 additional patients with other ( L tropica, L amazonensis, L braziliensis) types of cutaneous leishmaniasis this treatment was not effective. Infrared heat was studied in 178 patients with cutaneous leishmaniasis in Iraq, one application (55° C for 5 minutes) resulted in cure at 3 weeks in 162 (90%). Interestingly, they reported that other lesions that were untreated disappeared after treatment in 5-6 weeks.(19) In Israel, 18 patients (28 lesions) with acute cutaneous leishmaniasis were treated with localized ultrasound heating of lesions to 42° C for 2-3 minutes(0.5W/cm$^2$ to 3W/cm$^2$), twice to thrice per week for a total of 10-15 treatments. 13/18 patients showed complete resolution within 5-10 weeks of start of treatment (and 79% of lesions treated). Two persons with lesions on the face stopped early due to headache during and after heating.(20) Heat provided by radiofrequency may have some advantages. It evenly penetrates to a depth of about 4mm (depends on size of probe) so the upper dermis can be heated without injuring the underlying skin. The TTI Thermomed™ device uses localized current field radio frequency (a patented device based on technology developed at Los Alamos National Laboratory). The device directs current to a dual electrode surface stainless steel probe which is placed in skin contact. One arm of the probe has a thermocouple that monitors the temperature of the treatment site and controls the radiofrequency field to keep the temperature constant with a reported accuracy of ± 0.5 ° C. The Thermomed™ instrument is portable, battery operated. The technology does not transfer heat but generates heat within the tissue itself. (with diseased tissue apparently not tolerating the application of heat as well as healthy tissue- at least in malignancy) (21). The side effects are reported as "normal sloughing and oozing associated with healing and minimal scarring with no deformity." (TTI literature) Previous published studies of this treatment include Navin (22) randomly allocated 66 Guatemalan patients into placebo (sham application after local anesthesia), 15 days of 850 mg of antimony IM, and localized heat from radiofrequency wave 50 ° C for 30 seconds using 3 treatments at seven day intervals.(an earlier prototype device). The types of leishmaniasis were *Leishmania Viannia braziliensis* and *Leishmania Leishmania mexicana*. At 13 weeks after treatment beginning, complete healing with parasitologically negative lesions was 73% for antimony, 73% for localized heat and 27% for placebo. The only adverse event in the heat treatment was local infection (4 patients) despite the use of antibiotics concurrently. Authors advised that treatment of cellulitus before heat application may have benefit and reported that once they instituted this practice (late in protocol) no subsequent bacterial infections. Personal communication from Dr. Navin suggests that he had concerns about the

cosmetic effect (a sense that the scarring was more) and lesser healing as compared to glucantime and for this reason did not further pursue (note that he used a device that was a precursor to current one and photos in paper suggest much larger probe size). Velasco-Catrejon (23) showed that the device in a feasibility trial in Tabasco,Mexico had a cure rate of 90% at 8 weeks post treatment. This trial included 201 patients with *Leishmania mexicana* treated with a single application at 50 ° C for 30 seconds of whom 191 could be located for evaluation at 8 weeks. Dr. John David (Harvard Medical School) has presented unpublished data to us (March 17, 2003) from trials of the TTI ThermoMed™ device used in Brazil and a new study of 360 persons ongoing in Kabul Afghanistan since 9/2002. He related a treatment response of 80% from a single treatment and found the device field-expedient and easily transportable. He described no significant adverse events from heat therapy. He has observed resolution of other untreated lesions in about 1/3 patients and suggests that the killed parasite acts as a "self vaccine." In the Brazil trial, 37 patients with evaluation at 28 days (when crossed over to antimony) 71% cure with heat and 89% with glucantime 20mg/kg/20days. He noted decreased (*Leishmania*-stimulated peripheral blood mononuclear cells [PBMC]) Interleukin (IL)-10, IL-5, Interferon (IFN) gamma and Tumor Necrosis Factor (TNF) $\alpha$ at 28 days in both arms with no statistical differences. Also, lymphocyte proliferation and CD4/CD8 counts day 0,14,28 were similar (22) Besides the two trials mentioned above, a single patient was reported cured for *Leishmania tropica* cutaneous infection (he had 10 lesions, all but one responded to one treatment, one required 2 applications of heat) after treatment with a hand held radiofrequency heat generator (RDM Engineering Inc) at 50° Centigrade (C) for 30-60 seconds (24).

A rapid diagnostic polymerase chain reaction (PCR) test has been developed at WRAIR (25) Since the diagnosis of *Leishmania* species requires amplification in parasite culture the shipping to our contractor, Dr. Kreutzer in South Carolina, determination of *L major* will take about 4-6 weeks using our current technology; with a species (*L major*) specific primer in PCR this result can be effected in less than 24 hours. One of our investigators has been successful in developing species-specific *Leishmania* primers for PCR as have other scientists worldwide (26). In this study, prior obtained skin tissue for genus specific *Leishmania* PCR will also be subjected to the *L. major* specific primers PCR.

One of the challenging concerns, of local versus systemic treatment for cutaneous leishmaniasis, is the potential that this parasitic infection is systemic with manifestations in the skin, by time of diagnosis, with lymphatic involvement and potentially infected white blood cells that travel and can be associated with later complications, specifically mucosal/mucocutaneous leishmaniasis. For this reason, we will restrict our study to *L major*, which is clinically localized, unlike *L tropica* or the New World *Viannia* species. We will be able to compare the clinical response over one year of followup. However, we do not know if physical treatment with heat will give the same immunologic profile during and after treatment/cure as systemic treatment with Sodium stibogluconate. Preliminary presented data provided by Dr. David suggests that it may. We will incorporate study of the immunologic response in both arms pre, at about 10 days and at 6 months, through research assays using participant PBMCs and compare by treatment allocation as well as any that have a less than clinical cure by study arm.

**10. PLAN:**

**a. Investigational drugs/Devices status:**

Pentavalent antimony has been the mainstay of treatment for leishmaniasis for decades. It remains the drug of choice for cutaneous leishmaniasis in the U.S. Because of the limited need for Sodium stibogluconate in the United States, the Wellcome Foundation has not applied to the Food and Drug Administration (FDA) to license this drug in the US. Consequently, Sodium stibogluconate is available for use only under IND (Investigational New Drug) status (# 14150) currently held by the Department of the Army, Office of the Surgeon General. The Centers for Disease Control and Prevention (CDC) also has an IND for Sodium stibogluconate. Over 300 patients with leishmaniasis, primarily cutaneous but also mucocutaneous and viscerotropic, have been treated with Sodium stibogluconate at WRAMC since 1978 under protocols WU#1908,WU#1965,WU # 1978, WU 1993 and WU 01-19002. This represents the largest, single center experience with this disease and Sodium stibogluconate treatment in North America. We have detailed our experience with the currently recommended dose of 20mg/kg/day for 20 days (1)

The drug to be used in this study, Sodium stibogluconate, is investigational and will be used under IND number 14150, which is held by the Office of the Surgeon General. A copy of FDA Form 1572 is attached to this protocol application (appendix 2) and a copy of the Investigator's Brochure is attached (appendix 3) is on file in the Research Review Service, Department of Clinical Investigation.

The thermosurgery TTI ThermoMed™ device has been cleared by the FDA under a Section 510(k) premarket notification as being substantially equivalent to the legally market predicate devices marketed in interstate commerce prior to May 28,1976. The predicate devices are "electrosurgical cutting and coagulation devices and accessories" described in 21 CFR 878.4400 as "An electrosurgical cutting and coagulation device and accessories is a device intended to remove tissue and control bleeding by use of high-frequency electrical current". The FDA has classified these devices as Class II. ThermoMed™ was FDA cleared in 2002 for use in the treatment of cutaneous leishmaniasis as well as 15 other skin conditions (Appendix 1).

**b. Type and number of patients/charts/specimens to be studied:**

Up to 100 Military health care beneficiaries greater than or equal to 18 years of age presenting with the diagnosis of cutaneous leishmaniasis

**c. Inclusion and exclusion criteria:**

***Inclusion***:

1. Department of Defense (DOD) Healthcare beneficiary
2. Parasitologic diagnosis of cutaneous *Leishmania* infection **
3. Willing to locate to WRAMC area during treatment and perform subsequent follow up visits if needed*
4. Able to provide informed consent
5. All participants (both male and female) must agree to take precautions not to become pregnant or father a child for at least 2 months after receiving sodium stibogluconate

* If not active duty on orders, then the participant will bear the cost of food and lodging during the initial 10 day outpatient treatment period at Walter Reed Army Medical Center.

** Inclusion criteria for randomization includes that must be *Leishmania major* species

***Exclusion***:

1.Unable to provide informed consent

2. Pregnancy (females of child bearing potential must have negative urine Human chorionic gonadotropic hormone [HCG] within 48 hours start of infusion period)

3. History of hypersensitivity to Pentavalent antimonials

4.Serious medical illness

   a. QTc interval ≥ 0.5 sec
   b. severe cardiac disease
   c. history of recurrent pancreatitis
   d. liver failure or active hepatitis with transaminases > 3x normal
   e. renal failure or creatinine > 2.5
   f. thrombocytopenia (platelets <75,000)
   g. white blood cell count < 2000
   h. hematocrit < 25
   i. Absence of palpable extremity pulses in the limb requiring treatment

5. History of serious allergic reaction to local anesthetics
6. Location of lesion not amenable to local therapy (such as close to eye, mucous membranes, face, cartilage)
7. Presence of pacemaker and/or other implanted metallic devices
8. Breast Feeding
9. Men unwilling to avoid fathering a child during and/or in the two months following receiving the treatment
10. Women unwilling to avoid pregnancy for at least two months after receiving the treatment
11. More than 20 lesions, or multiple lesions which in the opinion of the investigator would not be well treated with heat therapy

Laboratory parameters in exclusion 4 are consistent with the WRAMC practice in prior and active Pentostam protocols as well as similar to guidelines used by the CDC for their IND. Laboratory results will be reviewed prior to initiation of Pentostam infusion.

**d. Recruitment:** Subjects will be referred after receiving a diagnosis of leishmaniasis by a treating physician. Infection with *L. major* species can be determined subsequent to recruitment. Subjects will be recruited, from those patients diagnosed with cutaneous leishmaniasis, by personal inquiry and discussion with potential participant by a study investigator. There will be no advertisement.

**e. Consent process:** Informed consent will be obtained by a discussion between a study physician investigator and potential subject prior to enrollment. This generally occurs as a one on one detailed discussion. Efforts will be taken to minimize undue influence. Potential subjects will be offered as much time they deem necessary to reach a decision, as well as the opportunity to discuss the protocol with others of their choosing. Privacy is provided for patient to discuss the protocol with investigator, to read the consent form without presence of study team, and to reach a decision. Consent process will be documented that the patient signed the informed consent prior to any study procedures being conducted and that the patient received a

signed copy of the protocol.

**f. Study design and methodology:**

This protocol will provide for the administration of the pentavalent antimonial, Sodium stibogluconate (Pentostam), or localized heat therapy for the treatment of patients diagnosed with cutaneous old world leishmaniasis. It is a prospective, randomized, IND study using sodium stibogluconate 20 mg/kg for 10 days as compared 1:1 to a single heat treatment of each *Leishmania* skin sore for 30 seconds at 50° C using a FDA cleared device (TTI ThermoMed™ instrument). All patients must be eligible for care at WRAMC. It is estimated that up to 100 subjects/27 in each treatment arm of study) will be enrolled in the first year. (Additional 46 included to allow for drop outs- mainly due to finding non L. major species). The length of study will be at least 5 years. The duration of the study for each participant will be one year.

**Subjects:** DOD health care beneficiaries diagnosed with cutaneous leishmaniasis, referred to the infectious disease service at Walter Reed Army Medical Center of age greater than or equal to 18 years, and any sex, gender,ethnicity, may be enrolled if study inclusion and exclusion criteria are met.

To receive treatment in this trial, the patient must have a definitive diagnosis of leishmaniasis. . Diagnosis will occur prior to enrollment and is not part of this protocol per se, except that patient must have definitive diagnosis to be eligible.

Establishment of the diagnosis will require one or more of the following: *Leishmania* culture confirmation (isolation of promastigotes in culture, ± characterization by isoenzyme pattern on electrophoresis), identification of *Leishmania* genus RNA using polymerase chain reaction, observation of amastigotes in histopathologically stained tissue, impression touch slides, or tissue scrapings (using Giemsa or hematoxylin and eosin staining). *Leishmania* cultures will usually be done in the College of American Pathologists (CAP) certified WRAIR Leishmaniasis diagnostic laboratory, histopathology will be done with review by Armed Forces Institute of Pathology (AFIP), generic *Leishmania* PCR will be done at WRAIR. These are all considered clinical tests.

> Diagnosis of *Leishmania major* infection will be defined as: + *Leishmania major* specific PCR of tissue sample and/or *Leishmania major* positive culture. It is anticipated that most participants will have previously had a skin biopsy for definitive diagnoses of leishmaniasis. If parasite culture and/or *Leishmania* PCR were not performed as part of that evaluation, then in this study, subjects will be asked to have another skin scraping or biopsy for *Leishmania* speciation which will be subjected to *Leishmania* culture at the Clinical Leishmania Diagnostics Laboratory (CAP-CLIA certified) and *L. major* specific PCR performed. If participant is not willing to provide a sample that allows parasite speciation then they can not meet inclusion criteria and will not be treated under this protocol. This parasite culture from skin sample is currently considered standard practice by the Leishmania Treatment Center and would be advised whether or not the patient chooses to participate in research study. If *L. major* infection can not be confirmed then patient can not proceed with treatment under this protocol.

Subjects will be identified by a six digit code, the first four digits being the year (2003,2004 etc) and the last two digits being random numbers.

**Description of the Protocol Drug:**
IND Number :14150
Sponsor: USAMMDA, Ft. Detrick, MD (will purchase and provide the Sodium stibogluconate)
Name of drug: Sodium stibogluconate (Pentostam) - comes as 100mg/ml solution in bottles of 100ml
Manufacturer: Wellcome Foundation, London UK
Storage of study medication: WRAMC clinical research pharmacy
Dose: 20mg/kg/day for 10 days
Concomitant medications allowed: All – patients agree to not use alcohol during treatment
Antidotes: The package insert notes that use of dimercaprol (200mg intramuscularly [IM] every 6 hours until recovery complete) and that 2,3 dimercaptosuccinic acid may also be an effective chelating treatment. If the patient receives an overdose, initiate intravenous hydration and move to ICU for cardiac monitoring. Consider obtaining serum sample at time of overdose recognition so that Sb level can be measured and collect 24 hour urine to measure excretion of Sb. (14)

Amount of Sodium stibogluconate to be used: The dose varies for each patient but on average 3 bottles (100 ml each) needed for every participant. Therefore 80 bottles in first year are estimated.

Once a diagnosis of Old World leishmaniasis is established, the patient will be offered enrollment and treatment as an outpatient. All eligible, enrolled patients randomized to the antimony treatment arm will receive Sodium stibogluconate . Sodium stibogluconate will be given by health care personnel at 20 mg/kg/day IV for 10 days. Infusion will generally occur in the WRAMC Infectious Disease [ID]clinic (or on the hospital ward if inpatient, or the infusion center if nursing support unexpectedly unavailable in the infectious disease clinic). If the infusion is given somewhere other than the ID clinic (such as the infusion clinic, an inpatient ward, or emergency room), then a study investigator will be present. Upon presentation of a prescription signed by a physician investigator, Sodium stibogluconate will be prepared in 50 ml Dextrose 5% water (D5W) by the WRAMC pharmacy service. It will be intravenously infused using butterfly needle or intravenous catheter over 30±20 minutes. It can be given intramuscularly if intravenous (IV) access is not possible, but this should be avoided for patient comfort unless necessary. For very obese persons, dosage may be modified and discussion with the principle investigator at (301)295-3621 and Dr. Barbara Herwaldt, CDC Division of Parasitic Diseases, at (770) 488-7760 is recommended. Sodium stibogluconate should be kept refridgerated when diluted. Sodium stibogluconate should be protected from light during storage and once diluted, however it is not necessary to cover it during the short infusion period.

Patients randomized to the heat therapy arm will be given one treatment using the ThermoMed™ Model 1.8 device at 50° C for 30 seconds each application, using a procedure (appendix 4) that gives overlapping heat applications to span the lesion and tissue directly beside it. This device will be obtained from Thermosurgery Technologies Inc in Phoenix AZ. This treatment will occur in either the Infectious Disease or Dermatology clinic WRAMC. Any patient having an appearance of a secondary bacterial infection of the skin lesion (common in skin ulcers) will be clinically

treated with an appropriate antibiotic as determined by an infectious disease physician until secondary infection is resolved **before** treatment with heat. For thermotherapy, the area around the lesion will be cleaned using betadine swabs, anaesthetised with 1% lidocaine HCl (without epinephrine), and moistened with sterile saline solution soaked gauze for 20±10 minutes prior to application of localised heat. Lesions < 2mm size will receive one application. All *Leishmania* skin lesions will be treated. There is a limit to 20 lesions that can be treated. For larger lesions treat in a linear fashion across the lesion with overlapping applications extending out to approximately 4 mm into apparently "healthy border skin". The probes that come in contact with participant skin will be cleaned after every usage as described in the user manual (appendix 4). The ThermoMed™ device will be loaned for use in this protocol through a no cost material examination agreement with Thermosurgery, Inc.

Randomization will done by the research pharmacist using a predetermined block randomization (provided by statistician) into one of two treatment groups. Group A will receive Sodium stibogluconate 20 mg/kg per day for 10 days and Group B will have one treatment of localized heat therapy using the ThermoMed™ device. Because of the obvious physical differences in treatment (10 days of IV treatment and one local heat treatment), no blinding will be done as the risk benefit to the patient was not in favor of sham treatments. Patients with clinical failure at the 2 months after treatment completion assessment will be offered crossover to the alternative treatment arm at about the 3 months after treatment timepoint.

Speciation of the Leishmania parasite will be done by PCR primer pairs in a research test, generally on tissue from a biopsy or tissue scraping done for diagnosis but not obtained specifically for this protocol. In some instances, the patient will have a parasite culture already completed and speciation with isoenzymes is accepted as evidence for L major.

Patients will be evaluated as an outpatient by a study physician. Participants will remain in the Washington D.C. area for the ten days of the interventional portion of the protocol (irregardless of randomization) Active duty participants will be sent in temporary duty status and generally stay in a nearby hotel. Individuals who are not active duty will be responsible for their own local housing and board. A germane baseline medical history will be obtained assisted by using a standardized data collection form. A physical examination (PE) will be done with special attention given to the nasopharynx, liver, spleen, lymphatics, extremity vascular supply, skin. A study investigator will see patients on days 0, 3±2 days, 7±2 days, 10±2 days. Toxicity will be monitored as listed in the table below and in protocol timeline table. Laboratory tests will be obtained prior to infusion/start of visit on each study day ±2 days. The WRAMC clinical laboratory will perform all routine assays.

All patients will have baseline studies of glucose,electrolytes, blood urea nitrogen (BUN), creatinine, alanine aminotransferase (SGPT), aspartate aminotransferase (SGOT), total bilirubin, alkaline phosphatase, amylase, lipase, creatine phosphokinase, complete blood count (hemoglobin, hematocrit, platelets, white blood cells with differential), and electrocardiogram[EKG](these will be considered screening studies which permit investigators to determine if patient can be enrolled in the treatment). Vital signs will be obtained prior to each Sodium stibogluconate infusion and during beginning of the daily visits in the heat treatment arm. Patients will be asked to report any

new symptoms to include local lesion pain, muscle pains, arthralgias, malaise, abdominal pain, anorexia, rash, palpitations. Follow up physical examinations will be conducted, directed by symptoms, on days 3±2 days, 7±2days, 10±2days.

**Lab Test Schedule**

| Day | EKG | CBC | CMP | CPK | Amylase | Lipase | PE | T cells | Immune tests | Total volume blood adult- (ml) |
|---|---|---|---|---|---|---|---|---|---|---|
| 1(Pre) * | X | X | X | X | X | X | X | X | X | 70.5 |
| 3 | X | X | X | X | X | X | X | | | 14.5 |
| 7 | X | X | X | X | X | X | X | | | 14.5 |
| 10 | X | X | X | X | X | X | X | X | X | 70.5 |
| 180 | | | | | | | | | X | 52 |

- Listed tests and EKG will be done anytime up to 48 hours prior to starting the study treatment

For female patients of child bearing potential, they should have a urine HCG test done within 48 hours before starting the Sodium stibogluconate infusion course. This is needed because of potential for unknown risk to the fetus. They will also have a urine HCG test on day 10 (±48 hrs).

Patients in both arms are being asked to have lab and EKG monitoring. In the heat treatment arm, some lab parameters are ordered to assess the degree of tissue destruction that occurs. The pancreatitis in treated leishmaniasis patients has been postulated to possibly be due to immune modulation during treatment, therefore it could apply to heat treatment as well so we will monitor this. The EKG monitoring was for comparison of toxicity between arms, as nonspecific T wave changes have frequently been seen in Sodium stibogluconate treated patients and not studied in heat treated patients.

Abnormalities in any of the monitored parameters may lead to interruption of the Sodium stibogluconate therapy, or to more frequent monitoring of indicated tests. Abnormal laboratories . and other adverse events (related to intervention) at the end of treatment period (day 10) will be assessed by a study investigator and recommendations and arrangements for followup by participant's local health provider will be made if deemed clinically required by the study physician investigator . Study investigators will follow with the local provider these adverse events until resolved or until termination of patient from protocol. Our usual clinical practice is to identify a follow up appointment on the clinic discharge paperwork and the WRAMC Tricare office makes the local appointment for the patient before he/she leaves the DC area. We provide each participant with information as to how to reach the principle investigator by phone,fax, email and mail. We initiate contact with participants at 2±1,6±1 and 12±1 months after treatment.

Photographs of cutaneous lesions prior to therapy and after treatment (day 9-12) and about 2 and 12 months after end of treatment (patients will be requested to provide and if they do not have a

camera then a disposable pocket camera will be distributed to them) will be obtained to document treatment status. Photos will be labeled only with patient study number and sequence (1= pretreatment, 2= about 10 days, 3= about 2 months after treatment complete and 4= about 12 months after treatment started). Patient will be given a measuring device to include in subsequent photos so diameter can be estimated. Photos are considered standard of care, to assist if potential relapse. At the end of study, photographs will be collected and scored for clinical response at 10±2 days, about 2 months after end of treatment and about 12 months after end of treatment, by an expert (Dr. Charles Oster) blinded to treatment allocation. Dr. Oster's role as investigator in this protocol execution will be limited to this.

Blood samples will be obtained for clinical monitoring as above and labeled with name and social security number with leftover samples discarded per Department of Pathology Area Laboratory Support [DPALS] (WRAMC) standard operating procedure. In addition, blood solely for immunologic research will be obtained in this study at baseline, about day 10 and about 6±1 months. The research blood will be labeled only with date and six digit study identifying number, no leftover blood is anticipated as we request the minimum to accomplish stated assays. The research blood will be taken by courier from the Infectious Disease clinic to WRAIR for further analysis. At the 6 month timepoint arrangements can be made to have blood (for immunologic assays) obtained at local treatment facility and express mailed to principal investigator. Immune function assays: T cell subsets will be obtained and done in the WRAMC immunology laboratory identified by personal health identifiers on day 0 and 10±96 hours. PBMC from pretreatment (Day 0), end of treatment (Day 10 ± 96 hours), and 6 months (± 4 weeks) will be obtained using six 8.5 ml yellow top tubes at each timepoint. One 10 ml green top will be added at the Day 0 and Day 10 timepoints (total volume is 182 ml over course of one year). The following assays are proposed:

1.Compare the antigen specific lymphoproliferative (LPA) and interferon-γ responses to *Leishmania* antigens before and after treatment in bulk PBMCs. In cells from participants with active lesions, the LPA and interferon γ will likely be reduced relative to those samples acquired when lesion is in a more healed state. With complete healing , the LPA and IFN- γ responses will likely increase.

Characterization of these regulatory T cells will be performed by FACS analysis of phenotype and intracellular cytokine production, mechanism of suppression via cytokine analysis. To assess the mechanism of suppression, will query what inhibitory cytokine produced (IL-10, IL5, versus TGF-β) using protein detection, Taqman for messenger RNA (mRNA) transcripts,and intracellular cytokine staining of the regulatory T cells.

Antigen will be the L *major* antigen from the Laboratory of Parasitic Diseases, National Institutes of Health [NIH] (Dr. Neva).

2. Examine host responses to *Leishmania* antigen in PBMCs pre and at day 10 timepoint using cDNA gene expression technology (methods further described in appendix 15).

Patients will not be required to physically come to Walter Reed for the 6±1 month timepoint. Arrangements to express mail the whole blood samples can be arranged by investigators with their local health care provider. Leftover blood is not anticipated as we have estimated the minimum

volume possible to complete these assays. Samples will be sent to the specimen processing facility at the Combined Military HIV Research Program in Rockville by courier (if patient local, by Federal express if patient returned to duty), where they will be stored until assayed identified as stated above. Because of collaborative HIV protocols, a Health Insurance Portability and Accountability Act (HIPAA) memorandum of understanding (MOU) between program and WRAMC is being developed.

Cutaneous leishmaniasis patients will be contacted for follow up at 2±1, 6±1 and 12±1 months post therapy. Contact will be by telephone, in writing via US mail,(appendix 6), email to provide the letter template, or patient may return to clinic for a visit. If lesions are reported as healed that information will suffice in conjunction with photo confirmation as cure. Any patient reporting recurrent lesions or relapse will be requested to return to WRAMC for evaluation. If patient reports interim significant (as determined by study investigator) medical evaluations or hospitalizations then copies of medical records will be requested.

Protocol Timeline

| Prior to Treatment | Day 1-10 | 2±1 months after treatment done | 6±1 months after treatment started | 12±1months after treatment started |
|---|---|---|---|---|
| L major PCR<br><br>Informed Consent<br><br>Baseline labs<br><br>Randomization<br><br>Photographs<br><br>Immune fn* labs | Study visits and labs per protocol<br><br>Initiate treatment<br><br>If SSG arm continue daily for 10 days<br><br>Day 10 photos<br><br>Adverse events<br><br>Day 10 immune fn labs | Photos<br><br>Adverse events<br><br>Eligible for crossover if clinical failure noted | Interval medical history<br><br>Immune fn labs<br><br>Adverse events | Interval medical history<br><br>Photos<br><br>Adverse events<br><br>Assign outcome<br><br>Volunteer registry database form |

*Immune function

**Outcome /Endpoint definitions:**

**-Clinical failure** for treatment of cutaneous leishmaniasis is defined as < complete re-epithelization and/or less than or equal to 75% reduction in the diameter of the skin lesion after starting a course of therapy as determined at the timepoint 2 months after completion of treatment. (this category will be offered crossover treatment)

**-Intermediate response** is less than complete re-epithelization and/or > 75% but less than 100% reduction in diameter of lesion at 2 months after completion of therapy and complete re-epithelization/reduction in lesion size at 12 months after start of therapy.

**-Clinical cure** for cutaneous lesions is complete epithelization (if ulcerative) and or greater than 75% reduction of size of lesion (if not ulcerative) at 2 months after completion of therapy and no reactivation up to 12 months after the start of therapy.

**-Relapse failure** is a patient who, regardless of appearance at 2 months after treatment completion, has activation of a consistent skin lesion either at the treatment site or elsewhere (if elsewhere parasitologic diagnosis will be confirmed) in the period up to 12 months after end of therapy.

In case of multiple skin lesions, each lesion will be scored for response.

Stopping rule: The major concern in this protocol is that of safety and toxicity. The efficacy of sodium stibogluconate is well described and ThermoMed device has equivalent efficacies in a large field trial in Afghanistan (another Old World parasite, L. tropica). ThermoMed is a FDA cleared device and the safety of its use has been cleared by the FDA. Sodium stibogluconate toxicity has been well described during its more than half a century usage. Toxicity can be evaluated within the ten day daily observation period. After the first seven secondary infections after treatment in the ThermoMed arm or after the first full thickness burn that requires surgical therapy or fails to heal in reasonable time -at the treatment site, then medical monitor will be queried, the protocol will be temporarily halted pending further evaluation and consideration given to stopping the protocol. The number of infections was estimated based on clinical experience, the prior published experience with ThermoMed, consideration that burned tissue is prone to infection, and anecdotally given that of the 35 patients currently on Pentostam, five are being treated for secondary infections- none of which were identified as present before initiation of treatment.

Crossover: For patients deemed to have clinical failure at 2 months, they will be offered crossover to the alternate treatment arm. This applies to participants that might not complete 10 days of Sodium stibogluconate due to toxicity (anticipated to be very rare), who are considered clinical failures at 2 months, and clinical failures in either treatment arm. There are 4 considered options at this point; crossover to other study treatment, continued observation without crossover, termination and enrollment in the standard 10-20 day course of sodium stibogluconate protocol, termination and treatment with FDA approved medications off label per clinical care. It is anticipated that most clinical failures will opt for the crossover option but other options can be available after primary endpoint reached. Criteria for this decision will include an assessment of the healing appearance of skin lesions, the patient's wishes, and physician recommendation. Those that elect to terminate will be asked to followup for 12 month study duration if willing.

**Risks/benefits:**

**Possible risks:**

During the process of drawing blood there may be some slight pain as the needle is inserted and a later swelling/bruise at site. Some people develop vasovagal responses to phlebotomy. The drug has several common characteristic side effects: about half of those receiving it complain of muscle and joint aches, some people lose their appetites. These side effects typically disappear shortly after the drug is discontinued. Sodium stibogluconate may cause irritation of the pancreas, typically shown by nausea, vomiting and abdominal pain (pancreatitis). Most patients may show high levels of pancreas irritation on blood tests during treatment. All cases of Sodium stibogluconate-induced pancreatitis, which have been identified at WRAMC, have improved once the drug was stopped. Our usual practice is to hold the Sodium stibogluconate for symptomatic pancreatitis until the symptoms have decreased and the pancreatic enzymes have declined about half of what they were at stopping. The risk of recurrence, if Sodium stibogluconate is resumed, is not known. In the cases, which were re-treated, no evidence of recurrent pancreatitis developed. Pancreatitis has the potential to be a serious, even life-threatening, illness. Those cases, seen at WRAMC, associated with Sodium stibogluconate use have all been mild. Other side effects include headache, feeling tired or weak, skin rash, irritation of the vein where IV is given, numbness or tingling in the hands or feet. There may be potentially serious, though reversible, adverse reactions that include injury to the liver and kidneys, and abnormal heart rhythms. These are rare (<2%) side effects. Other side effects: Sodium stibogluconate may cause a lowering of blood counts including a severe decrease in the number of platelets circulating in the blood (thrombocytopenia). Thrombocytopenia may result in bleeding of varying severity. All patients with Sodium stibogluconate-induced decreased platelets, identified so far, have had prompt return of their platelets to normal counts, without bleeding, after stopping Sodium stibogluconate. Like all drugs, it is possible that a person could be allergic to Sodium stibogluconate and have an anaphylactoid reaction. To my knowledge, this has not seen in the previous 25 years of use at WRAMC.

The ThermoMed™ Model 1.8 device, (a localized current field radiofrequency instrument), delivers heat at high temperature to the skin. Because diseased tissue doesn't seem to tolerate heat as well as healthy skin, the infected tissue may be killed before the normal tissue is. An expected side effect of this treatment is a burn and potentially blistering at the site of treatment. The treatment causes burn pain which is why local numbing medicine is given first. However after the treatment, for several days, there may be discomfort at the site of treatment which feels like a skin burn. Previously unknown allergy to the lidocaine could be severe, even life threatening, if not reversed quickly. If a bacterial overgrowth of the leishmaniasis lesion is present, then antibiotics would be given first to avoid the treatment causing worsening of the bacterial infection. For blistering burns, topical treatment, such as silvadene may be advised. Volunteers may slough off the top skin and have some oozing from the treatment site for up to 14 days as the area heals. A burn may cause a scar as it heals, but so may leishmaniasis. According to the company and other doctors who have used the ThermoMed™ device this is not seen very often. Keloid- makers may develop a keloid at the site of the heat treatment.

Efforts to minimize the risk of sodium stibogluconate include attendance during infusion by a study investigator, frequent monitoring with physician visits, laboratory tests and EKG, screening for pregnancy pre treatment and having a crash care and epinephrine available in the area of

infusion. As above, for the heat treatment antibiotics will be used if secondary infection apparent, and topical treatment may be used if blistering burn occurs. Local anesthetic will be used to minimize discomfort. As before, supplies needed to manage cardiac arrhythmias, allergic reactions are available in the treatment room of the infectious diseases clinic. An allergy history will be obtained in the baseline medical history inquiry. In addition, ammonia inhalant is available for those who feel faint during venipuncture. Pressure bandage will be applied to the site after venipuncture. If a skin scraping or biopsy is done to obtain a culture and speciation after enrollment (anticipated to be unusual), then sterile technique will be implemented after using lidocaine local anethesia. After the procedure a bandage will be placed over the site. No sutures will be used as the size of a biopsy will generally be 4mm or less.

**Possible benefits:**

Participant will receive the drug of choice but for a shorter time than currently recommended (because of findings from a recent study published by us) or a FDA cleared heat treatment to treat their Leishmaniasis infection, with very careful monitoring for side effects. The possible benefit from participation in this study is cure of *Leishmania* infection. However, no benefit can be guaranteed. The information gained may improve the understanding of the course of the infection and the immune response to treatment.

**Plan for monitoring and assessing safety:** The IND sponsor (USAMMDA) will provide monitoring for the protocol in accordance with 21CFR312. Routine monitoring will occur at least annually. Safety parameters include healing of lesion, evaluation for complications of therapies such as anaphylaxis, pancreatitis, prolonged QT interval on the EKG, chemical hepatitis, musculoskeletal complaints, decreased blood counts, complication of pregnancy in the time surrounding drug administration. SAEs per written protocol definition will be collected and reported whether felt related or not to study intervention. Participants will be seen daily by a physician, and all infusions and device treatments will be attended by a study investigator. There are also frequent investigator visits during the 10 interventional days and follow-ups at 2,6,12 months. Healing of cutaneous infection can easily be identified by appearance. A stopping rule has been included in this protocol. Secondary complications such as bacterial superinfection or burn can be treated with antibiotics or silvadene. Allergic reactions will be monitored for by study investigator and clinic doctors, who will have all resources of a tertiary medical care facility (WRAMC), a crash cart, epinephrine and ammonia inhalant available in the treatment room to respond to allergic and vasovagal events. Pregnancy will be tested for pre and post 10 day intervention and participants will be counseled to avoid pregnancy during this time. Frequent laboratory testing and EKG during intervention phase will screen for early changes allowing response of study investigators to temporarily hold, stop, increase monitoring, depending on their clinical judgement.

**Withdrawal Criteria:** Volunteers will be allowed to withdraw from the study at any time without prejudice or loss of benefits to which they are entitled. Volunteers may be removed from the study by a study investigator or the medical monitor should their continued participation be considered injurious to their health and well being at any time. Factors for discontinuation that can be anticipated from our prior experience would be symptomatic pancreatitis that does not respond to a drug interruption or recurs on rechallenge with sodium stibogluconate, moderate

activity limiting arthritis, severe arthralgias, platelet count < 50,000, QTc prolongation more than 500, liver function tests more than 10 times normal limits, anaphylaxis related to infusion of sodium stibogluconate, urticaria termporally related to infusion of sodium stibogluconate. The WRAMC Human use Committee (HUC) will be informed of each withdrawal through the annual progress report. Volunteers withdrawing from study after receiving any Pentostam or heat treatment will be requested to continue with follow ups at 2, 6 and 12 months. It is not anticipated that withdrawn volunteers will be replaced. Since the study is of relatively modest duration, the dropout rate during the initial treatment phase is expected to be quite low. However, our experience suggests that up to 20% of active duty individuals may be difficult to track for 6 and 12 month follow-ups due to deployments and moves.

**g. Serious and unexpected adverse events:**

The recording of adverse events is the responsibility of the investigators. Volunteers will be instructed to contact the investigators promptly in the event that they develop any new signs or symptoms during the clinical trial.

An adverse event temporally related to participating in the study should be documented whether or not it is considered to be related to the test article. This definition includes intercurrent illnesses and injuries and exacerbations of preexisting conditions. Any adverse reaction which occurs must be noted and recorded on the appropriate case report forms. Those that are identified in the protocol ("expected") should be forwarded to sponsor and Institutional Review Board (IRB) during annual report. Unexpected (but not serious) adverse events possibly related to participation in the protocol should be reported to the Human Use Committee, WRAMC, within 10 working days (see below for Medical Research and Materiel Command [MRMC] policy).

A serious adverse event is an untoward medical occurrence that at any dose:

1. Results in persistent or significant disability/incapacity
1. Is a congenital anomaly/birth defect
2. Is associated with a new cancer diagnosis
3. Overdose
4. Results in death
5. Is life-threatening
6. Requires inpatient hospitalization or prolongs existing hospitalization

Any serious or unexpected adverse event must be reported immediately by telephone to USAMRMC Deputy Chief of Staff for Regulatory Compliance and Quality (301) 619-2165 and a serious adverse event (SAE) form must be faxed to (301) 619-7803. The study sponsor representative (MAJ David Shoemaker at (301) 619-7871) must also be notified as well as the Human Use Committee, WRAMC within 24 hours. A written report addressed to U.S. Army Medical Research and Materiel Command ATTN: MCMR-RCQ, 504 Scott Street, Ft. Detrick, MD 21702-5012 must follow in 3 working days. SAEs will be reported to the medical monitor (Dr. Kester at (301) 319-9234) within 3 working days. In the SAE report, include name of reporting person, name of study, HSRRB log number, work unit number, number of patients enrolled to date, and number and type of serious and unexpected adverse events previously reported in the study as well as a description of the current event. In addition include the subject identification number and initials, associate investigators name and the name of the medical treatment facility (MTF), subjects date of birth, gender and ethnicity, test article and dates of administration, signs and symptoms and severity, date of onset, date of resolution or death, relationship to the study drug, action taken, concomitant medications including dose, route, and duration of treatment, and date of last dose. * Serious adverse events must be reported even if the investigator believes that the event is unrelated to the protocol agent.

The principal investigator (PI) within one working day must report all serious adverse events occurring in subjects enrolled at WRAMC to the Human Use Committee (HUC). This is accomplished by submitting an adverse event report memorandum to the HUC via Department of Clinical Investigation (DCI). For protocols involving investigational drugs or devices, the investigator must also report a serious adverse event to the sponsor of the IND or Investigational Device (IDE) immediately (within 24 hours).
Unexpected (but not serious) adverse events occurring in subjects enrolled at WRAMC which, in the opinion of the PI, are possibly related to participation in the protocol must be reported by the PI within 10 (ten) working days to the HUC using the same procedure.
Expected adverse events, (i.e., those events included as potential risks in the consent form) which are not serious, should be reported yearly on the Annual Progress Report (APR) for each protocol. A summary of all serious or unexpected side effects also must be included in the APR. If there were no adverse events, this must be stated on the APR.

Follow up for adverse events will include tracking them with participant for resolution up to 12 months. Abnormal laboratories at the end of treatment period (day 10) will be assessed by a study investigator and recommendations and arrangements for followup by participant's local health

provider will be made if deemed clinically required by the study physician investigator. Serious adverse events will be monitored to resolution/stability or for the duration of the protocol, which ever applies. Participants will be offered appropriate level of care in military treatment facilities for their adverse events, and when needed will be returned to WRAMC for specialty care. Consultation by study investigator with their treating provider will occur for any SAE felt to be potentially related to the study intervention or Leishmaniasis.

**h. Human Biological Specimens:**

Specimens will be sent to a non-WRAMC facility for analysis; in both cases to the WRAIR laboratory of WRAMC credentialed physicians who bridge both clinical and laboratory. The tissue includes skin biopsy tissue (obtained using surgical permit routine at WRAMC for invasive procedures) that was obtained prior to enrollment and may have already been extracted for genus Leishmania PCR. Remaining tissue or Leishmania RNA may be further analyzed using TAQman probe for species level Leishmania diagnosis (this is considered a research test whereas genus level PCR is a clinical test). Other obtained tissue is blood, specifically PMBCs which are obtained with informed consent in this protocol and processed in the laboratory of Dr. Marovich and Dr. Peel. No leftover PBMC are expected and solely the immune responses to treatment of leishmaniasis will be studied. Access to PBMCs will be limited to Dr Marovich, Dr. Peel and their technical staff and the principal investigator. As listed in protocol, samples for research testing will be labeled with a date collected (day 0, day 10, day 180), study number, patient identifying number. Immune function testing will include a profiling of genes that are turned on and off during this treatment using a microarray containing 9182 non-redundant sequence verified genes analyzed using a laser scanner, GenPix 4000A. It is important to note that we are not measuring changes in the persons' genetic makeup, rather we are measuring RNA transcripts that act to regulate either positively or negatively the thousands of proteins made in response to infection. The samples will only be kept until the assays are completed. Shipping of samples at 6 months when participant may be back at home station will be by federal express to the Principle investigator who will then insure that all identifiers potentially included on samples are removed and labeling for lab as above. Samples collected at WRAMC will be sent by courier service that picks up three times per day on ward 63 (in place to support HIV research studies) and delivers to WRAIR. No future use is planned. Participants may withdraw from lab studies if they notify PI prior to assays being performed and the samples will be destroyed .

i. **Patient confidentiality**: Study records will be maintained in a locked file cabinet in a locked room located on Ward 63, Walter Reed Army Medical Center. Source document files and Case report files will be identified with the patients six digit code.

The study investigators, medical monitor, representatives of WRAMC Department of Clinical Investigation, Uniformed Services University of the Health Sciences Office of Research, the Human Subjects Research Review Board, the Army Clinical Investigation Regulatory Office, U.S. Army Medical Research and Material Command, the U.S. Food and Drug Agency, and other government agencies as part of their duties, may access study data to monitor, audit, IRB review and pursue regulatory inspections of the source data and study documents as part of their

responsibility to protect human subjects in research. Final study data, in the format of a manuscript, will be shared with Thermosurgery Technologies, Inc.

**Disposition of Data:**

A copy of the protocol, protocol regulatory records, case report forms, source documents will be kept on file by the Infectious Disease Service, Walter Reed Army Medical Center (WRAMC) for at least 10 years after completion of study. Per DoD regulation, the records will need to be archived for 75 years. Future disposition of records after this time would include shredding to maintain confidentiality.

Please see appendix 13 which includes a HIPAA authorization form.

1. Are you intending to collect data on any of the 18 personal health identifiers?
___ No – HIPAA does not apply – go to question #4
_x__ Yes – please check which ones:

X 1. Names
_X 2. Street address, city, county, 5-digit zip code
_X 3. Months and dates (years are OK) and ages >89 (unless all persons over 89 years are aggregated into a single category)
_X_4. Telephone numbers
___ 5. Fax numbers
_X_6. E-mail addresses
_X 7. Social security number
___ 8. Medical record number
___ 9. Health plan beneficiary number
___ 10. Account number
___ 11. Certificate/license number
___ 12. Vehicle identification number (VIN) and/or license plate number
___ 13. Device identifiers and serial numbers
___ 14. URLs (Uniform Resource Locators)
___ 15. Internet protocol address number
___ 16. Biometric identifiers, such as finger and voice prints
___ 17. Full face photographic images or any comparable images
___ 18. Any other unique identifying number, characteristic, or code such as patient initials

2. Can you limit your collection of personal health identifiers to just dates, city/state/zip, and/or "other unique identifier" (#18 above)?
___ Yes – then your dataset may qualify as a Limited Data Set – please complete a Data Use Agreement and attach to your protocol. Then go to question #4.
_X__ No – Go to question #3.

3. Is obtaining patient Authorization "impracticable"?
___ Yes – Authorization may qualify to be waived by the IRB. Provide a detailed justification why you believe obtaining an Authorization is impracticable. (If the Waiver is approved and your sample size is less than 50, disclosures of data outside the Military Healthcare System must be tracked by the PI.)
_X__ No – Research subjects will need to sign a HIPAA Authorization. Complete the

Authorization and attach to this protocol.

4. What precautions will you take to protect the confidentiality of research source documents (Case Report Forms, questionnaires, etc.), the research datafile, and the master code (if any)?
See above confidentiality section
5. When will you destroy the research source documents, datafile, and the master code?
See above data disposition section
6. Will research data with any personal health identifiers be sent outside of WRAMC?
_X__ Yes – Please explain assurances you have received from the outside party that they will appropriately follow confidentiality protections, follow the HIPAA requirements, and abide by the provisions of your Authorization. Samples will be sent to associate investigator labs at WRAIR who by signing this protocol will be demonstrating their assurance to follow requirements delineated herein
___ No.

j. **Data collection**: Data will be collected using case report forms (appendix 7). In addition a data sheet (recipe) will be used to follow protocol requirements during the infusion phase (appendix 8). Data collected will include epidemiologic information, medical history, physical examination, pre and post photographs, toxicity monitoring, immunologic studies, laboratory results and EKGs. In addition, follow up information about clinical outcome (cure) will be sought. A completed copy of the Volunteer Registry Database form (Appendix 9) will be submitted to USAMRMC, ATTN: MCMR-RCQ, 504 Scott Street, Ft. Detrick, MD 21702-5012 once a patient is terminated from the study.

It is the policy of USAMRMC that data sheets are to be completed on all volunteers participating in research for entry into the U.S. Army Medical Research and Materiel Command Volunteer Registry Database. The information to be entered into this confidential database includes name, address, social security number, study name, and dates. The intent of the database is twofold; first, to readily answer questions concerning an individual's participation in research sponsored by the USAMRMC; and second, to ensure that the USAMRMC can exercise its obligation to ensure research volunteers are adequately warned (duty to warn) of risks and to provide new information as it becomes available. The information will be stored in the USAMRMC for a minimum of 75 years.

**k. Sample size estimation:**
The study will be analyzed on an intent to treat basis. A sample size of 27 patients per treatment group was planned based on the assumption that the cure rate in the heat arm will be 73% (Navin et. al, 9) compared to 99% cure rate in the sodium stibogluconate arm (Wortmann et al., 6) Controlling for the probability of a Type I error at alpha = 0.05, sample of 27 subjects per group will have 80% power to detect a 26% difference. To allow for dropouts up to 100 subjects can be entered into the study.

l. **Data analysis**: Cure rates and toxicity outcomes will be compared using the Fishers exact test. Endpoints for patients that are crossed over will be presented descriptively. For all measured

laboratory variables, data at days 0,7,10 will be compared between groups by use of repeated measures analysis of variance. To satisfy assumptions of normality and sphericity for this method, the natural logarithm of each variable was analyzed. Clinical response will be determined at the end of the protocol by a blinded evaluator who will not know what treatment was received by each subject. Photographs will be provided at baseline, 10 days, 2 months and 12 months for determination of endpoint. Missing data will be managed in the analysis as missing and all attempts will be made to avoid missed visits at 2 and 12 month after treatment. Analysis will be done both by each lesion response as well as by each participant.

A study database will be maintained by the principal investigator using a SPSS or Access format where participants are identified by study identifying number without personal health information.

**Quality Assurance:**
The QA section of USAMMDA will monitor this study.

**Accountability Procedures for the Investigational Product and Medications Storage:**
Pentostam (Sodium stibogluconate) will be delivered from manufacturer to Division of Experimental Therapeutics, Walter Reed Army Institute of Research, drug repository. On request of study investigator (POC is Dr. Bill Ellis), IND Study medication will be transferred to the Walter Reed Army Medical Center research pharmacy. The research pharmacy will maintain a written and operating procedure for the inventory, dispensing, accounting, preparation of the study drug. Unused study drug that has expired or is deemed non-usable can be disposed of by the research pharmacy with notification in advance to the principal investigator.

**Management of Protocol Deviations:**
A log of protocol deviations will be maintained by the principal investigator. Deviations that might (in the opinion of the PI) cause potential harm to subject will be reported to the medical monitor (Dr. Kester) ,the WRAMC Human use Committee and the HSRRB.

**Modifications to Protocol:**
Modifications/addenda to this protocol will be submitted in written format for review to the WRAMC Department of Clinical Investigation (DCI). Investigator will forward all addenda to Human Subjects Research Review Board (HSRRB) for second level review, however action can be taken after receipt of primary level (WRAMC) approval. Any modifications made to the protocol which will increase the level of risk to the subject, will also be submitted to the Human Subjects Research Review Board for approval prior to initiation. Copies of addenda and IRB approval letters will be forwarded to sponsor and USUHS Office of Research.

**Ethics**
The risks to volunteers associated with participation in this study are generally reversible, and the benefit to society in developing a shorter and less toxic regimen to treat cutaneous Leishmaniasis (one of 5 leading tropical diseases worldwide) is high. The proposed regimens have a significant pre- test likelihood from prior studies to offer treatment benefit to the individual participant. The volunteers will not be compensated for their participation. Progress on this protocol will be

provided to the WRAMC Human Use Committee annually and as appropriate during the study. Copies of approved annual progress reports will be provided to the HSRRB and USUHS IRB.

**Publication Policy:**
Results of this study will be presented in scientific forums orally and in written format in peer reviewed scientific journals. Issues related to authorship that can not be resolved among the investigator team, will be referred to an ad hoc committee of the Chief, Infectious Diseases WRAMC, a representative of WRAMC Department of Clinical Investigation, and the USAMMDA product manager for decision.

## 11. REFERENCES:


1. Aronson NE, Wortmann GW, Johnson SC, Jackson JE, Gasser RA Jr, Magill A et. al. Safety and efficacy of intravenous sodium stibogluconate in the treatment of leishmaniasis: Recent U.S. military experience. Clin Infect Dis 1998; 27(6):1457-64.

2. Pathak MK, Yi T. Sodium stibogluconate is a potent inhibitor of protein tyrosine phosphatases and augments cytokine responses in hemopoetic cell lines. J Immunol 2001; 167(6):3391-7.

3. Ballou WR, Gordon DM, McClain JB et al. Safety and efficacy of high dose sodium stibogluconate therapy of American Cutaneous Leishmaniasis. Lancet 1987; i:13-16.

4. Oster CN, Chulay JD, Hendricks LD. et al. American Cutaneous Leishmaniasis: A comparison of three sodium stibogluconate treatment schedules. Am J Trop Med Hyg 1985; 34(5):856-860.

5. Herwaldt BL and JD Berman. Recommendations for treating Leishmaniasis with sodium stibogluconate (Pentostam) and review of pertinent clinical studies. Am J Trop Med Hyg 1992; 46(3):296-306.

6. Wortman G, Miller RS, Oster C, Jackson J, Aronson N. A randomized, double-blind study of the efficacy of a 10 or 20 day course of Sodium stibogluconate for treatment of cutaneous leishmaniasis in U.S. military personnel. CID 2002; 35:261-7.

7. Anabwani GM, Ngira JA, Dimiti G et al. Comparison of two dosage schedules of sodium stibogluconate in the treatment of visceral leishmaniasis in Kenya. Lancet 1983; i:210-213.

8. Thakur CP, Kumar M, Kumar P et al. Rationalisation of regimens of treatment of kala-azar with sodium stibogluconate in India: a randomised study. Br Med J 1988; 296:1557-1561.

9. Navin TR, Arana BA, Arana FE et al. Placebo-controlled clinical trial of sodium stibogluconate (Pentostam) vs. ketoconazole for treating cutaneous leishmaniasis in Guatemala. JID 1992; 165:528-534.

10. Saenz RE, de Rodriguez CG, Johnson CM et al. Efficacy and toxicity of Pentostam against Panamanian mucosal leishmaniasis. Am J Trop Med Hyg 1991; 44:394-398.

11. Franke ED, Wignall FS, Cruz ME et al. Efficacy and toxicity of sodium stibogluconate for mucosal leishmaniasis. Ann Intern Med 1990; 113:934-940.

12. Chulay JD, Spencer HC, Mugambi M. et al. Electrocardiographic changes during treatment of leishmaniasis with pentavalent antimony (sodium stibogluconate). Am J Trop Med Hyg 1985; 34:702-709.

13. Thakur CP. Harmful effect of high dose stibogluconate treatment of kala-azar in India. Trans R Soc Trop Med Hyg 1986; 80:672-3.

14. Herwaldt BL, Kaye ET, Lepore TJ et al. Sodium stibogluconate (Pentostam) overdose during treatment of American Cutaneous Leishmaniasis. JID 1992; 165:968-71.

15. Sacks DL, Barral A, Neva F. Thermosensitivity patterns of old versus new world cutaneous strains of Leishmaniasis growing within mouse peritoneal macrophages in vitro. Am J Trop Med Hyg 1983. 32(2): 300-304.

16. Berman J, Neva F. Effect of temperature on multiplication of Leishmania amastigotes within human monocyte-derived macrophages in vitro. Am J Trop Med Hyg 1981; 318-321.

17. Pereira C, de Castro MP, de Mello D. Influencia da temperature cutanea no desenvolvimiento de lesoes leishmanioticas. Arq Inst Biol (São Paulo) 1958; 25:121-138.

18. Neva FA, Petersen EA, Corsey R, Bogaert H, Martinez D. Observations on local heat treatment for cutaneous leishmaniasis. Am J Trop Med Hyg 1984; 33(5):800-804.

19. Junaid AJN. Treatment of cutaneous leishmaniasis with infrared heat. Int J Derm 1986; 25:473-475.

20. Aram H, Leibovici V. Ultrasound-induced hyperthermia in the treatment of cutaneous leishmaniasis. Cutis 1987; 40:350-353.

21. Kainer RA, Stringer JM, Lueker DC. Hyperthermia for treatment of ocular squamous cell tumours in cattle. JAVMA 1980; 176(4):356-360.

22. Navin TR, Arana BA, Arana F et al. Placebo-controlled clinical trial of meglumine antimoniate (Glucantime) vs. localized controlled heat in the treatment of cutaneous leishmaniasis in Guatemala. Am J Trop Med Hyg 1990; 42(1):43-50.

23. Velasco-Castrejon O, Walton BC, Rivas - Sanchez B et al. Treatment of cutaneous leishmaniasis with localized current field (radio frequency) in Tabasco, Mexico. Am J Trop Med Hyg 1997; 57(3):309-312.

24. Levine N. Cutaneous leishmaniasis treated with controlled localized heating. Arch Dermatol 1992; 128:759-761.

25. Wortmann G, Sweeney C, Houng HS, Aronson N, Stiteler J, Jackson J, Ockenhouse C. Rapid diagnosis of leishmaniasis by fluorogenic polymerase chain reaction. Am J Trop Med Hyg 2001; 65(5):583-587.

26. Hochberg L, Ryan J, Arana B, et al. Diagnosis and subgenus identification of cutaneous leishmaniasis in Guatemala and Panama using Smart Cycler® technology. Abstracts of the 51st annual meeting of the Am Soc Trop Med Hygiene, page 185, Denver, CO, 2002.

## 12. FACILITIES/ORGANIZATIONS TO BE USED:

The patient will receive Pentostam infusions and protocol assessments in the infectious disease clinic, hospital wards, infusion center, or emergency room of Walter Reed Army Medical Center(WRAMC). The localized heat treatment will be applied in either the Infectious Disease Clinic or the Dermatology clinic, (WRAMC). The WRAMC research pharmacy will maintain and dispense the study drug. The *Leishmania major* PCR will be performed by Dr. Wortmann and the immune function assays by Dr. Marovich and Dr. Peel using laboratory facilities at the Walter Reed Army Institute of Research, Silver Spring MD and Rockville MD.

## 13. ROLE AND RESPONSIBILITIES OF EACH INVESTIGATOR AND COLLABORATOR:

The medical monitor will be required to review all serious adverse events and unexpected adverse events associated with the protocol and provide an unbiased written report of the event within 10 calendar days of the initial report. At a minimum the medical monitor should comment on the outcome of the adverse event and relationship of the event to the test article. The medical monitor should also indicate whether he/she concurs with the details of the report provided by the PI. The medical monitor will forward their report to the US Army Medical Research and Materiel Command,ATTN: MCMR-RCQ, 504 Scott Street, Fort Detrick MD 21702-5012.

The principal investigator responsibilities are catalogued later in this document.

All associate investigators will be responsible for recruitment, assessment of study inclusion/exclusion criteria, enrollment, treatment administration, completion of protocol study visits and pertinent case reports, review of study laboratories, immediate reporting of SAEs and unexpected adverse events (UAEs) to the principle investigator (and in her absence to the above described boards/offices). Dr. Oster will be primarily responsible for the scoring of the photographs and will not perform patient visits. Dr. Wortmann will be also responsible for performing the *Leishmania major* PCR and providing results back to

study investigators. Dr. Marovich will be responsible for performing the immunologic assays.

The collaborating investigator will be responsible for performing the microarray studies of gene expression pre and at day 10±96 hours timepoint.

**I. Clause 13.01 - Responsibilities of the Principal Investigator to The Surgeon General (TSG)Through the USAMRMC, Office of the Deputy Chief of Staff**

a. To promptly report changes or unanticipated problems in a research activity. Normally, changes may not be initiated without The TSG approval, except where necessary to eliminate apparent immediate hazards to the human subject or others.

b. To immediately report by telephone (DSN 343-2165 or 301-619-2165) (non-duty hours call 301-619-2165 and send information by facsimile to DSN 343-7803 or 301-619-7803) adverse experiences that are both serious and unexpected.* For those projects involving an Investigational New Drug (IND) application sponsored by TSG, a written report will follow the initial telephone call within 3 working days.

c. To promptly report any change of investigators.

d. To prepare, at a minimum, an annual progress report or final report in accordance with Title 21, Code of Federal Regulations, Part 312.33.

e. To immediately report by telephone (DSN 343-2165 or 301-619-2165) knowledge of a pending compliance inspection by the Food and Drug Administration (FDA) or other outside governmental agency concerning clinical investigation or research.

---------------------------------------------------------------

*"Serious adverse experience" means any experience that suggests a significant hazard, contradiction, side effect, or precaution. With respect to human clinical experience, a serious adverse drug experience includes any adverse drug experience that is fatal or life-threatening, is permanently disabling, requires inpatient hospitalization, or is a congenital anomaly, cancer, or overdose.

"Unexpected adverse experience" means any adverse experience that is not identified in nature, severity, or frequency in the current investigator brochure; or, if an investigator brochure is not required, that is not identified in nature, severity, or frequency in the risk information described in the general investigational plan or elsewhere in the current application, as amended.

## 14. <u>TIME REQUIRED TO COMPLETE:</u>

**Anticipated start date – JAN 2004**
**Expected completion date – AUG 2008**

## 15. BUDGET:

**Will any outside organization provide funding or other resources? Yes ( ) No ( X )**

USAMMDA will provide Sodium stibogluconate at the cost to WRAMC of $1615.00 as well as provide study monitoring for a 5 year total cost of $59,574.29. These costs have been authorized to be paid to USAMMDA by NARMC command and it is anticipated that the protocol may be completed well before 5 years at the current rate of leishmaniasis cases from Iraq.

Most of the costs of this protocol are those for the needed clinical care of patients with leishmaniasis. Research costs are for the species specific PCR and the immunologic studies.

The ThermoMed device will be loaned under a no cost evaluation for future purchase agreement with Thermosurgery Technologies Inc, Phoenix AZ.

The WRAMC Infectious Disease Service will provide a nurse coordinator, clinical supplies such as intravenous supplies, blood drawing equipment, betadine, sterile normal saline, lidocaine, gauze, folders for case report forms (CRFs) and source documents, a locked file cabinet for records. ID service digital camera can be used for in house photographs. Film processing (mail in cameras) will be provided by Audiovisual (AV) service WRAMC.

**DCI Budget Request for Intramural Protocols Only:**

| | **FY03** | **FY04** | **TOTAL** |
|---|---|---|---|
| **Consumable Supplies** (Itemize each supply) | $1500 | $3000 | $4500 |
| **Other*** | | | |
| **Travel**** | | | $1000 |
| TOTAL *** | $1500 | $3000 | $5500 |

## 16. ENVIRONMENTAL IMPACT STATEMENT:

For Questions contact: Maged Abdel-Rahim, Chief, Research Operations Service, DCI at (202) 782-7612

Does any part of this protocol generate hazardous chemical waste as defined by Title 40 of the Code of Federal Regulation YES ( ) NO ( X )? If yes, at what stages and how much?

Have you considered any alternative procedures? YES ( ) NO ( X) If yes, why were

they rejected?

**17. <u>INVESTIGATOR COMPLIANCE</u> with AR 40-38, Clinical Investigation Program; WRAMC Regulation 70-1, WRAMC Research Activities; and WRAMC Federal-Wide Assurance (FWA):**

a. I have read and will comply with AR 40-38, Clinical Investigation Program, and WRAMC Regulation 70-1, WRAMC Research Activities.

b. I have read and will comply with the Health Services Command memorandum of 7 December 1987, Subject: Clinical Investigation Funds.

c. I have read and will comply with WRAMC Federal-Wide Assurance granted by the Office for Human Research Protection, Department of Health and Human Services.

d. I certify that any outside funds and/or other resources (other than requested from DCI) being provided for this study are listed above in this application under <u>Budget</u>.

**18. <u>RESPONSIBILITIES OF THE PRINCIPAL INVESTIGATOR IN HUMAN SUBJECTS RESEARCH</u>:**
**The principal investigator is the individual who is primarily responsible for the actual execution of the clinical investigation. He/she is responsible for the conduct of the study, obtaining subjects' consent, providing necessary reports, and maintaining study documents. The principal investigator:**

1. Will not enroll a subject into a study until the study has been approved by the appropriate authority and, when appropriate, the subject's primary care physician has granted approval for him/her to enter a study.

2. By signing this protocol, I warrant that any use of Protected Health Information for reviews preparatory to research met the following requirements:
a. The review of Protected Health Information was done solely to prepare a research protocol, or for similar purposes preparatory to research;
b. No Protected Health Information was taken outside the Military Health System; and
c. This review of PHI was necessary for research purposes

3. Is responsible for assuring that the prospective volunteer is not participating as a subject in other research that will significantly increase the research risks.

4. Is responsible for assuring the quality of each subject's consent in accordance with current federal regulations. This will include ensuring that any "designee" that obtains consent on your behalf is completely conversant with the protocol and is qualified to perform this responsibility.

5. Will obtain the appropriate WRAMC /HSRRB clearance for advertisements used to recruit

research subjects.

6. Will not accept any outside personal remuneration for implementation of a study.

7. Will take all necessary precautions to ensure that the study does not generate hazardous chemical waste.

8. Will obtain the proper WRAMC clearance for all presentations, abstracts, and publications. The following require WRAMC approval:

   a. Reports involving WRAMC patients.
   b. Reports that cite WRAMC in the title or byline.
   c. Reports of WRAMC approved clinical investigation or research.
   d. Reports of research performed at WRAMC.
   e. Reports of research conducted by WRAMC assigned personnel.

9. Must submit to the Department of Clinical Investigation WRAMC/HSRRB:

   a. Any source of outside funding.
   b. An Annual Progress Report (APR) due in the anniversary month of the protocol's initial approval.
   c. Reports of adverse effects occurring in subjects as a result of study participation.
   d. An Addendum, if changes need to be made to the study design or number of patients to be enrolled.
   e. A Final Report within 30 days following termination of a study.
   f. A listing of presentations, abstracts, and publications arising from the study for inclusion in the DCI Annual Research Progress Report.

10. Will maintain a Study File that must be kept for three years following completion of the study if no IND/IDE used (32 CFR 219.115(b). If IND medication or IDE appliances are used, the file must be kept for 2 years after FDA approval and can then be destroyed; or if no application is filed or approved, until 2 years after the study is discontinued and FDA notified (21CFR 312.62(c). The records should be kept in the Department/Service where the research took place (AR 40-38). If you PCS or ETS, these records should be given to a new Walter Reed PI or the Department/Service Chief.

    This file may be inspected at any time by DCI, the Clinical Investigation Regulatory Office (CIRO), HSRRB, sponsor, the Food and Drug Administration (FDA), and/or other regulatory agencies responsible for the oversight of research. This file will include:

    a. The approved protocol and applicable addenda.
    b. The approval memorandum and WRAMC Clinical Investigation and Human Use Committee minutes (as appropriate) granting approval to initiate the study.
    c. Other applicable committee minutes [e.g., Radioactive Drug Research Committee (RDRC); the Surgeon General's Human Subjects Research Review

Board].

d. Each Volunteer Agreement Affidavit (DA 5303-R) signed by the subject and a Witness.
e. Annual Progress/Final Reports.
f. Reports of adverse effects occurring in subjects as a result of study participation.
g. Reports of any significant new findings found during the course of the study.
h. All study documents generated from study date.
i. Publications, abstracts, reprints resulting from study data.
j. All information pertaining to an investigational drug or device.
k. For HIV research studies, approval of the Chief, Infectious Disease Service.

11. Will be familiar with all applicable regulations governing research, and will adhere to all of the requirements outlined in the WRAMC Federal-Wide Assurance granted by the Office for Human Research Protections, Department of Health and Human Services.

The undersigned Principal and Associate investigators have reviewed this protocol and will conduct the study in full compliance with Good Clinical Practices and the Army's regulations.

**19. PRINCIPAL INVESTIGATOR'S SIGNATURE: With my signature I as the Principal Investigator acknowledge that I have read the responsibilities and will comply with them. I understand that if I fail to comply with any of these responsibilities, all projects for which I am an investigator may be suspended. I also acknowledge the above Application for Clinical Investigation Project; Request for Approval of Clinical Investigation Study Proposal; Environmental Impact Statement; Investigator Compliance Statement; and Responsibilities of the Principal Investigator in Human Subject Research.**

Naomi E. Aronson

PRINCIPAL INVESTIGATOR
COL Naomi Aronson
Director, Infectious Diseases Division USUHS
Infectious Diseases/Medicine

**20. ASSOCIATE INVESTIGATORS' SIGNATURE:**

Gail Robinson

Associate Investigator
MAJ(RET) Gail Robinson
Head Nurse, Leishmania Treatment Center
Infectious Diseases/ Medicine

______________________________

Associate Investigator
LTC Glenn Wortmann
Associate Program Director, Infectious Diseases Fellowship
Infectious Diseases/ Medicine

Associate Investigator
COL Paul Benson
Staff Dermatologist
Dermatology/Medicine

Associate Investigator
Dr. Mary Marovich
Senior Scientist, Combined Military HIV Research Program
Henry M. Jackson Foundation/WRAIR

Associate Investigator
MAJ Mark Polhemus
Fellow, Infectious Diseases
Infectious Diseases/Medicine

Associate Investigator
COL Charles Oster
Chief, Infectious Disease Clinic
Infectious Disease Service/Medicine

Associate Investigator
COL Wendy Bernstein
Staff Scientist
Retrovirology, WRAIR

Associate Investigator
MAJ Mark Polhemus
Fellow, Infectious Diseases
Infectious Diseases/Medicine

Associate Investigator
COL Charles Oster
Chief, Infectious Disease Clinic
Infectious Disease Service/Medicine

Wendy B. Bernstein COL MC
Associate Investigator
COL Wendy Bernstein
Staff Scientist
Retrovirology, WRAIR

[illegible]

**20. OTHER SIGNATURES for APPROVAL:**

1. I have reviewed this proposal with the investigator for:
   a. Scientific merit
   b. Experimental design
   c. Expenditure of money and man-hours
   d. Risks and safeguards in the use of human subjects
2. This proposal and its impacts is forwarded with my full support and approval.

[signature] 5 May 03
SERVICE CHIEF
LTC(P) Clifton Hawkes MC
C, Infectious Diseases
Department of Medicine

[signature] [illegible]
DEPARTMENT CHIEF
COL William Duncan MC
C, Department of Medicine
WRAMC

[signature] 9 May 03
MEDICAL MONITOR
LTC (P) Kent Kester MC
C, Clinical Trials
WRAIR

[signature]
Dr. Robert Goldstein
Chair, Department of Medicine
USUHS

May 14 2003
Version

**Aronson, Naomi E COL WRAMC-Wash DC**

**From:** Wortmann, Glenn W LTC WRAMC-Wash DC
**Sent:** Wednesday, May 21, 2003 11:58 PM
**To:** Aronson, Naomi E COL WRAMC-Wash DC
**Subject:** RE: NEW PROTOCOL

Thanks. I have reviewed the heat vs pentostam for the treatment of cutaneous leishmaniasis protocol and concur to participation as an AI.

-----Original Message-----
From: Aronson, Naomi E COL WRAMC-Wash DC
To: Wortmann, Glenn W LTC WRAMC-Wash DC
Sent: 5/21/03 10:53 AM
Subject: NEW PROTOCOL

Please accept this - LTC Wortmann currently in Afghanistan but should return by JUL 03

Associate Investigator
MAJ Mark Polhemus
Fellow, Infectious Diseases
Infectious Diseases/Medicine

Associate Investigator
COL Charles Oster
Chief, Infectious Disease Clinic
Infectious Disease Service/Medicine

## 21. OTHER SIGNATURES for APPROVAL:

**I concur with the submission of this proposal to the Clinical Investigation Committee and/or Human Use Committee for review and approval.**

SERVICE CHIEF
LTC(P) Clifton Hawkes MC
C, Infectious Diseases
Department of Medicine

DEPARTMENT CHIEF
COL William Duncan MC
C, Department of Medicine
WRAMC

MEDICAL MONITOR
LTC (P) Kent Kester MC
C, Clinical Trials
WRAIR

Dr. Robert Goldstein
Chair, Department of Medicine
USUHS

suspended. I also acknowledge the above Application for Clinical Investigation Project; Request for Approval of Clinical Investigation Study Proposal; Environmental Impact Statement; Investigator Compliance Statement; and Responsibilities of the Principal Investigator in Human Subject Research.

PRINCIPAL INVESTIGATOR
COL Naomi Aronson
Director, Infectious Diseases Division USUHS

Infectious Diseases/Medicine

20. ASSOCIATE INVESTIGATORS' SIGNATURE:

________________________________
Associate Investigator
MAJ(RET) Gail Robinson
Head Nurse, Leishmania Treatment Center

Infectious Diseases/ Medicine

________________________________
Associate Investigator
LTC Glenn Wortmann
Associate Program Director, Infectious Diseases Fellowship

Infectious Diseases/ Medicine

________________________________
Associate Investigator
COL Paul Benson
Staff Dermatologist
Dermatology/Medicine

Mary A. Marovich
________________________________
Associate Investigator
Dr. Mary Marovich
Senior Scientist, Combined Military HIV Research Program

Henry M. Jackson Foundation/WRAIR

________________________________
Associate Investigator
MAJ Mark Polhemus
Fellow, Infectious Diseases
Infectious Diseases/Medicine

________________________________
Associate Investigator
COL Charles Oster
Chief, Infectious Disease Clinic

**20. OTHER SIGNATURES for APPROVAL:**

1. I have reviewed this proposal with the investigator for:
   a. Scientific merit
   b. Experimental design
   c. Expenditure of money and man-hours
   d. Risks and safeguards in the use of human subjects
2. This proposal and its impacts is forwarded with my full support and approval.

5 May 03 | 9 May 03 | 9 May 03
---|---|---
SERVICE CHIEF | DEPARTMENT CHIEF | MEDICAL MONITOR
LTC(P) Clifton Hawkes MC | COL William Duncan MC | LTC (P) Kent Kester MC
C, Infectious Diseases | C, Department of Medicine | C, Clinical Trials
Department of Medicine | WRAMC | WRAIR

Dr. Robert Goldstein
Chair, Department of Medicine
USUHS

May 18, 2003
Naomi Aronson
Director, Infectious Diseases Division USUHS

ARCH PROGRAM

RE: Leishmaniasis study

**Protocol Title: Phase I/II randomized comparison of localized heat therapy versus Sodium Stibogluconate for the treatment of Old World cutaneous leishmaniasis**

13 Taft Court
Suite 200
Rockville, Maryland
20850
Tel: 301-251-5000
Fax: 301-762-4177

Dear Naomi,
I am very enthusiastic about our collaboration on this leishmaniasis study. Our immunology laboratory is planning to support this study in the following ways:

1. Process the blood from the subjects to obtain peripheral blood mononuclear cells (PBMCs) for further study. The PBMCs will be frozen and studied later (batched) when all the related specimens can be run together for optimal control.
2. Prepare the purified T cells subsets using Dynal beads (or suitable substitute) as detailed in the study.
3. Stimulate the cells in lymphocyte proliferation assays (bulk assays and depletion studies) and collect the supernatants and calculate the proliferation induced by the antigens. The supernatants will be analyzed for cytokine production (protein detection). If warranted, other methods to detect cytokines will be performed such as Taqman to measure RNA transcripts and intracellular cytokine production (flow cytometry techniques).
4. Phentoypic characterization of T cell subsets using flow cytometry with standard monoclonal antibodies-fluorochrome conjugates.
5. If a skin biopsy is obtained and the subject is willing, we can perform similar cellular characterization of the lesion (and the evolution of it at various stages of healing) using simple immunostaining techniques.
6. We will cover the shipping costs (Fed Ex) for the specimens.

I am looking forward to this collaboration to further our understanding of immunologic changes in leishmaniasis and in response to different therapeutic modalities: both in the active setting and various stages of healing.

Very respectfully submitted,

Mary A. Marovich, M.D., DTM&H
WRAIR/HMJF
13 Taft Ct., Suite 200
Rockville, MD 20850
Ph 301 251-8337, Fax: 301 762-4177

DEPARTMENT OF THE ARMY
WALTER REED ARMY INSTITUTE OF RESEARCH
WALTER REED ARMY MEDICAL CENTER
WASHINGTON, D.C. 20307-5100

REPLY TO
ATTENTION OF

RECEIVED 6/16/03

09 June 2003

COL Naomi E. Aronson, MD, MC
Director, Infectious Diseases Division
Department of Medicine
Uniform Services University of Health Sciences

Dear Naomi,

This letter is to confirm my willingness to collaborate with you to use microarray technology to advance research initiatives in the treatment of Old World cutaneous Leishmaniasis. As you know, my laboratory is employing this technology to advance research efforts in HIV-1 pathogenesis, *Plasmodium falciparum* co-infection models, and host response to infection. My laboratory contains all equipment necessary for fabrication of de novo microarrays including high throughput PCR capacity, a Chip Writer Pro microarrayer, a GenPix 4000A non-confocal laser scanner, and bioinformatic software for imaging and analysis of microarray data. Efforts to develop an innovative, robust web-enabled database for archival of de novo microarray data, a novel search engine for sophisticated queries of microarray data across aggregate experiments, and an analysis information management system for relevant mathematical transformations of microarray data are nearly complete and will contribute substantially to our ability to examine gene expression profiles to host responses to Leishmanaial antigens.

Best of luck with your grant proposal. Please do not hesitate to contact me if I can be of any additional assistance.

Best regards,

Dr. Sheila A. Peel
Director De Novo Microarrays/Flow Cytometry
Depart. Molecular Diagnostics/Pathogenesis
Div Retrovirology
Walter Reed Army Institute Research
1600 East Gude Dr.
Rockville, MD 20850
301-251-8346 (phone/voicemail)
301-762-7460 (fax)
speel@hivresearch.org (e-mail)
